# Single-cell transcriptomic characterization of microscopic colitis

**DOI:** 10.1038/s41467-025-59648-8

**Published:** 2025-05-18

**Authors:** Stefan Halvorsen, Molly Thomas, Mari Mino-Kenudson, Yuko Kinowaki, Kristin E. Burke, David Morgan, Kaia C. Miller, Katherine M. Williams, Jenny Gurung, Jessica McGoldrick, Megan Hopton, Brooke Hoppe, Nandini Samanta, Sidney Martin, Alice Tirard, Benjamin Y. Arnold, Jessica Tantivit, Joseph Yarze, Kyle Staller, Daniel C. Chung, Alexandra-Chloé Villani, Slim Sassi, Hamed Khalili

**Affiliations:** 1 Center for Computational and Integrative Biology, Massachusetts General Hospital (MGH), Boston, MA USA; 2 Center for Immunology and Inflammatory Diseases, Department of Medicine, MGH, Boston, MA USA; 3 Krantz Family Center for Cancer Research, Department of Medicine, MGH, Boston, MA USA; 4 Broad Institute of Massachusetts Institute of Technology (MIT) and Harvard, Cambridge, MA USA; 5 Harvard Medical School (HMS), Boston, MA USA; 6 Division of Gastroenterology, Department of Medicine, MGH, Boston, MA USA; 7 Department of Pathology, HMS, MGH, Boston, MA USA; 8 Clinical and Translational Epidemiology Unit, MGH, Boston, MA USA; 9 Department of Orthopedic Surgery, MGH, Boston, MA USA; 10 Institute of Environmental Medicine, Nutrition Epidemiology, Karolinska Institutet, Stockholm, Sweden; 11 Present Address: Department of Medicine, Duke University Health System, NC Durham, USA

**Keywords:** Transcriptomics, Gastrointestinal diseases, Chronic inflammation, Mucosal immunology, Lymphocytes

## Abstract

Microscopic colitis (MC) is a chronic inflammatory disease of the large intestine and a common cause of chronic diarrhea in older adults. Here, we use single-cell RNA sequencing analysis of colonic mucosal tissue to build a cellular and molecular model for MC. Our results show that in MC, there is a substantial expansion of tissue CD8^+^ T cells, likely arising from local expansion following T cell receptor engagement. Within the T cell compartment, MC is characterized by a shift in CD8 tissue-resident memory T cells towards a highly cytotoxic and inflammatory phenotype and expansion of CD4^+^ T regulatory cells. These results provide insight into inflammatory cytokines shaping MC pathogenesis and highlight notable similarities and differences with other immune-mediated intestinal diseases, including a common upregulation of *IL26* and an MC-specific upregulation of *IL10*. These data help identify targets against enteric T cell subsets as an effective strategy for treatment of MC.

## Abstract (web summary)

Microscopic colitis is a chronic inflammatory disease of the large intestine. Here the authors use single-cell RNA transcriptomic profiling and tissue localization studies to characterise the colon immune cell populations in MC, showing expansion of CD8 T cells with diverse TCR clonotypes and expression of CD4 T reg cell signatures.

## Introduction

Microscopic colitis (MC) is an increasingly recognized chronic inflammatory disease of the large intestine that primarily affects older adults and is associated with increased mortality^1^ and decreased quality of life^2,3^. MC is one of the most common causes of diarrhea and fecal incontinence in older adults and is associated with several commonly used medications such as proton pump inhibitors, non-steroidal anti-inflammatory drugs, and selective serotonin reuptake inhibitors^4–7^. The burden of MC has increased significantly over the past few decades^8–10^, likely due to an aging population, polypharmacy, and increased disease awareness. Nevertheless, the pathophysiology of MC remains poorly understood, and currently, there are no FDA-approved treatments for the disease.

A significant challenge in studying MC has been the remarkably similar macroscopic mucosal appearance to healthy colonic mucosa^7^, necessitating a histological examination for diagnosis. Diagnostic criteria include greater than 20 intraepithelial lymphocytes (IELs) per 100 epithelial cells in the lymphocytic colitis subtype, and increased IELs with a thickened collagen band ( ≥ 10 μm) in the collagenous colitis subtype^11^. These expanded IELs were previously identified to be predominantly CD8^+^ T cells^12,13^, although the pathogenic mechanism driving the expansion and inflammatory phenotype is unclear. Single-cell RNAseq (scRNAseq) has been used to identify key mediators of disease progression in other gut inflammatory disorders, such as checkpoint inhibitor-induced colitis (irColitis)^14,15^ and ulcerative colitis (UC)^16^. Here, we use scRNAseq to define the cellular and molecular perturbations in the colon mucosa of patients with MC compared to unaffected controls and patients with chronic diarrhea.

## Results

### All major colonic cell types are represented

**Fig. 1:**
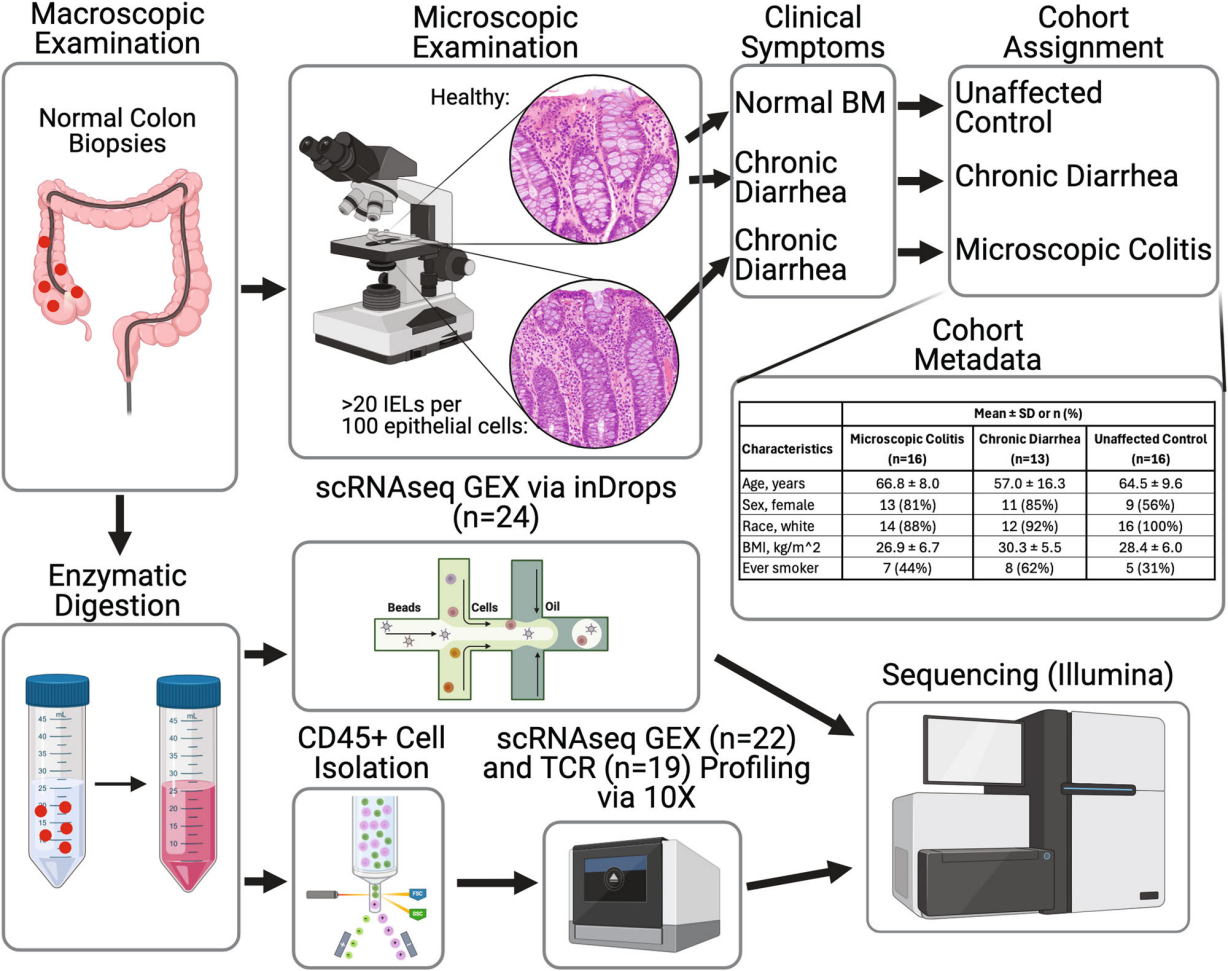
Study overview. Overview of the study design, sample collection, and sequencing. Patients were classified into three cohorts based on a combination of microscopic examination of colon biopsies and clinical symptoms (*n* = 16 for active microscopic colitis, *n* = 13 for chronic diarrhea, and *n* = 16 for unaffected controls). Biopsies were enzymatically digested, and single cells were encapsulated using either 10X Genomics or inDrops technologies. Library construction was finished, and libraries were sequenced on Illumina instruments. Created in BioRender. Halvorsen, S. (2025) https://BioRender.com/d85k894.

**Fig. 2:**
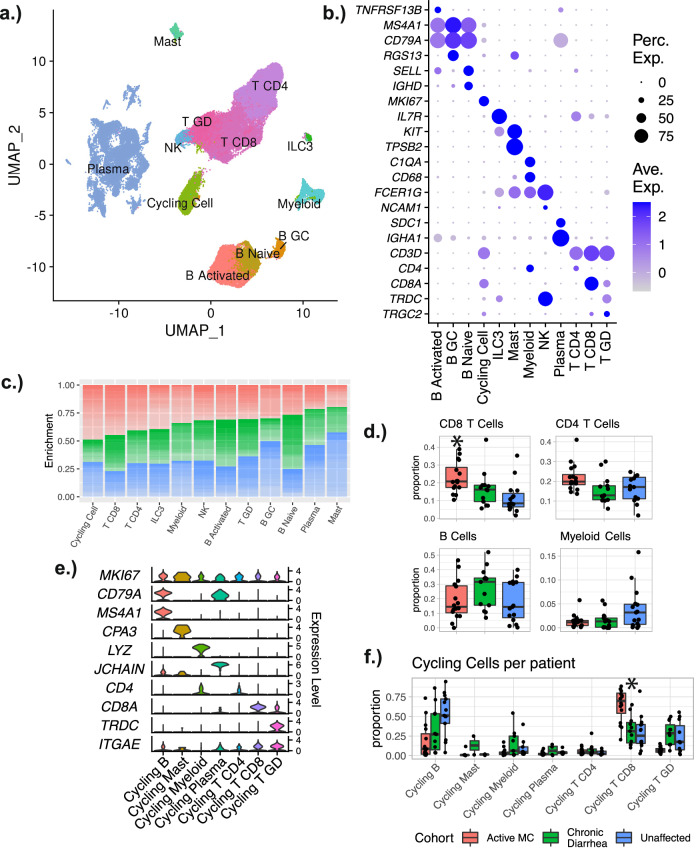
Overview of immune cells. **a** UMAP plot illustrating all immune cells and corresponding cluster assignments. **b** Dot plots showing the distribution of representative markers for the cell types. **c** Stacked bar plot demonstrating the relative enrichment of each cell type by cohort designation. **d** Boxplots showing the per-patient proportional differences from selected cell types. The proportions shown are the proportions of all immune cells for each patient. **e** Violin plots showing the distribution of representative markers for the different types of cycling cells. **f** Boxplots showing the per-patient proportional differences from the different types of cycling cells. The proportions shown are relative to the number of all cycling cells for each patient. For (**D**) and (**F**) significance was calculated using scCODA—n.s. indicates the MC proportions are not significantly different from controls, and an asterisk (*) indicates significance at an FDR level of 0.05. Number of patients in each cohort in each panel: MC (*n* = 16); chronic diarrhea (*n* = 13); unaffected (*n* = 15). Patients with no cells in a given identity class were not plotted. Boxplot center line represents the median; the box bounds span from the first to the third quartile; whiskers extend from the box to the largest value no further than 1.5 * inter-quartile-range from the box. Source data are provided as a Source Data file.

Endoscopic colon mucosal biopsies were collected from 16 individuals with symptomatic biopsy-proven MC, 17 people undergoing screening colonoscopy, and 13 individuals undergoing diagnostic colonoscopy for chronic diarrhea with normal histologic findings. scRNAseq libraries from these samples were generated using either the inDrops^17^ (*n* = 7 for MC, 6 for chronic diarrhea, 10 for unaffected controls, 1 for asymptomatic MC) or 10X Genomics platforms (*n* = 9 for MC, 7 for chronic diarrhea, and 6 for unaffected controls). An overview of patient demographics and methods is shown in Fig. 1, and metadata for all patients in the final dataset is shown in Supplementary Data 1. After quality control, 132,381 cells were recovered. After batch correction and clustering (Supplementary Fig. S1), cells were annotated by cross-referencing identified markers with published literature^14,16,18^ (Fig. 2a, b). All cellular subsets were represented in multiple patients (minimum = 12, average = 35 patients, Supplementary Fig. S2), indicating a high level of reproducibility. All major immune cell populations were captured, including rarer cell types such as plasmacytoid dendritic cells (pDCs, *n* = 56 across 16 patients) and granulocytes like neutrophils (*n* = 83 across 27 patients), which are not readily captured with 10X chemistry^16^ (Fig. 2a–c, Supplementary Figs. S2, 3). Captured epithelial cells include the major expected populations, covering enterocytes, transit amplifying (TA), enteroendocrine, goblet, and tuft cells^16^ (Supplementary Fig. S4). The stromal dataset captured fibroblasts, endothelial, fibroblast-like^19^, and glia cells^20,21^ (Supplementary Fig. S4). Of note, epithelial, stromal, and granulocyte datasets were mostly generated using the inDrops platform because the 10X libraries were generated from CD45^+^ sorted cells (excluding granulocytes). The low number of epithelial and stromal cells in our dataset limited our ability to resolve and analyze smaller cellular subtypes that have been previously described^16^.

### Tissue cytotoxic CD8 T cells expand in microscopic colitis

Our in-depth analysis centered on immune cells because the diagnosis of MC is based on the colonic mucosal expansion of lymphocytes. Among the major immune lineages, the most significant shifts in cell abundance were observed in the CD8 T cell and plasma cell compartments (Fig. 2c, d). CD8 T cells were strongly enriched in MC, while plasma cells (*SDC1*^Hi^ and *IGHA1*/*JCHAIN*^Hi^ or *IGHG1*^Hi^) were depleted. Prior immunohistochemistry (IHC) based^12,13^ studies of immune cells in MC noted an increase in CD8^+^ T cells. The decrease in plasma cells, however, was unexpected and is in contrast to conventional histologic assessment of MC^7,22^. Interestingly, a similar pattern was observed in prior scRNAseq studies of UC and irColitis^14,16^. Further, compared to controls, patients with MC present with higher ratios of IgG (*IGHG*) to IgA (*IGHA*) expressing plasma cells (Supplementary Fig. S5G). The decrease in plasma cells could be a technical artifact, as the decrease was most pronounced in the inDrops data (freshly dissociated tissue) as compared to the 10X genomics data (cryopreserved cells) (Supplementary Fig. S6A). CD4 T cells, B cells, and Myeloid cells presented no significant proportional alterations associated with MC (Fig. 2d).

### MC exhibits a shift towards activated tissue-resident memory CD8 T cells

**Fig. 3:**
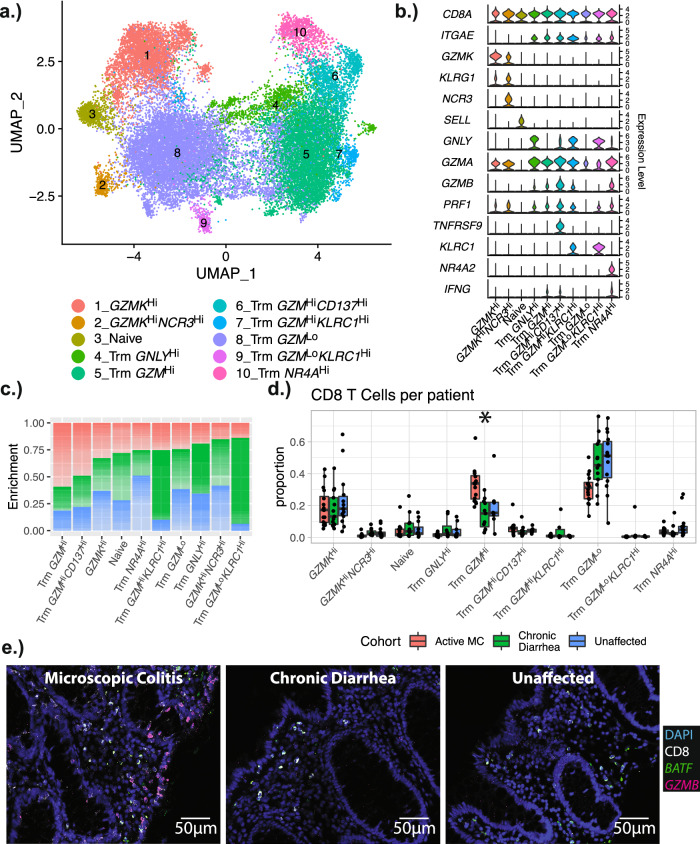
Subclustering of CD8 T Cells. **a** UMAP plot showing the subclustering of CD8 T cells, and corresponding cluster designations. **b** Violin plots showing the distribution of representative markers for the identified cell types. **c** Stacked bar plot demonstrating the relative enrichment of each CD8 T cell subset by cohort designation. **d** Boxplots showing the per-patient proportional differences from each of the subtypes. The proportions shown are relative to the number of all CD8 T cells for each patient. Significance was calculated using scCODA—n.s. indicates the MC proportions are not significantly different from controls, and an asterisk (*) indicates significance at an FDR level of 0.05. Number of patients in each cohort: MC (*n* = 16); chronic diarrhea (*n* = 13); unaffected controls (*n* = 15). Patients with no cells in a given identity class were not plotted. Boxplot center line represents the median; the box bounds span from the first to the third quartile; whiskers extend from the box to the largest value no further than 1.5 * inter-quartile-range from the box. **e** RNAscope images of a representative patient from each cohort. Number of slides examined for each cohort: *n* = 8 for active MC, *n* = 8 for CD, *n* = 9 for unaffected controls. The fluorescent images are colored as follows: DAPI: blue; CD8: white; *BATF*: green; *GZMB*: magenta. Quantitation of all patient slides and individual channels is shown separately in Supplementary Fig. S11. Source data are provided as a Source Data file.

CD8 T cells exhibited the strongest expansion of any immune cell type in MC, so this population was sub-clustered and examined in detail (Fig. 3). Cell subtypes were identified as naïve (*SELL*^Hi^ and *CCR7* ^Hi^), *GZMK*^Hi^ cells, and multiple types of *ITGAE*^Hi^ (i.e. CD103^+^) tissue-resident memory (Trm) T cells (Fig. 3a, b). CD103 is a marker for Trm cells, but recent reports have identified subpopulations of Trms that do not express CD103^23,24^, indicating that the *GZMK*^Hi^ T cells could also represent a CD103^-^ Trm population. One subset of *ITGAE*^Hi^ Trm cells expressed *NR4A* family transcription factors, the transcriptional regulators *EGR2* and *EGR3*^25–27^ and activation markers such as *CD69*. These genes are all stimulated upon TCR engagement^25–31^. Expression of effector genes, such as the granzymes A and B, was used to define cytotoxic cell states among *ITGAE*^Hi^ Trm cells^32,33^ (cells with high levels of *GZMA* and *GZMB* were labeled as *GZM*^Hi^ cells, while cells with low levels of *GZMA* and *GZMB* were labeled as *GZM*^Lo^ cells). One activated *ITGAE*^Hi^ population was marked by high expression of *CD137*, a TNF receptor with strong TCR co-stimulatory activity in activated T cells^34^.

Sub-clustering analysis revealed a decrease in non-activated *GZM*^Lo^ *ITGAE*^Hi^ Trm cells and a corresponding increase in activated *GZM*^Hi^ *ITGAE*^Hi^ Trm populations in MC patients (Fig. 3c, d). These patterns were consistent across patients and independent from the scRNAseq profiling method used (inDrops or 10X), supporting the robustness of this observation (Supplementary Fig. 6). These results suggest a global shift in the CD8 *ITGAE*^Hi^ Trm populations away from a resting phenotype and towards a more activated and cytotoxic phenotype in MC. The activated *ITGAE*^Hi^ Trm populations show marked upregulation of *IFNG*, which corresponds with the increased interferon gamma (IFN-G) in patients with MC^35,36^ (Fig. 3b, Supplementary Fig. S7).

### In MC, cytotoxic CD8 T cells are distributed throughout lamina propria and epithelial compartments

IHC and RNAscope^37^ were used to assess the tissue localization of CD8^+^ T cell populations enriched in MC. CD8 and GZMB were first stained using quantitative IHC. CD8^+^ T cells were significantly increased in patients with MC in both the lamina propria and epithelial compartments (Supplementary Fig. S8). GZMB staining intensity was weak but detectable in some cells (Supplementary Fig. S9). Although the counts were low, GZMB^+^ cells were significantly higher in patients with MC.

RNAscope was then used to visualize and quantify the expression of a selection of upregulated genes identified with a pseudobulk differential gene expression analysis. The top MC-associated genes were filtered based on abundance, log-fold-change, and probe availability; *HLA-DRB1, LINC02446, BATF*, and *GZMB* were chosen for staining. The RNAscope slides were counter-stained with an antibody against CD8. Expression of *HLA-DRB1*, *LINC02446, BATF*, and *GZMB* were increased in both the epithelial and lamina propria compartments in patients with MC (Fig. 3e, Supplementary Fig. S10, S11). While low levels of *GZMB* (1, 2 copies per cell) were detected in many CD8^+^ T cells from both MC cases and controls, MC samples had significantly higher levels of *GZMB* compared to controls (Fig. 3e, Supplementary Fig. S11). The increased expression of *HLA-DRB1* was ubiquitous, but most pronounced in the epithelial compartment (epithelial cells and infiltrating IELs) (Supplementary Fig. S10, S12). *LINC02446* exhibited similar staining patterns as *HLA-DRB1*, although the increased epithelial layer expression was not nearly as striking as *HLA-DRB1* (Supplementary Fig. S10). In contrast to the widespread epithelial compartment and lamina propria expression of *HLA-DRB1* and *LINC02446*, *GZMB* and *BATF* were predominantly localized to CD8^+^ T cells (Supplementary Fig. S11). Using an automated pipeline to quantify lamina propria cells (see “RNAscope staining and analysis” methods section), expression of CD8, *HLA-DRB1*, *LINC02446, GZMB*, and *BATF* was modeled and found to be significantly higher in MC cases than controls. Collectively, these histology data are indicative of an activated and inflammatory cell state in MC that persists across both the lamina propria and epithelial compartments.

### Increased abundance of CD8 T Cells in MC is likely due to local expansion

Cycling CD8 T cells (*MKI67*^Hi^ or *PCNA*^Hi^) were substantially enriched in MC compared to unaffected controls (Fig. 2e, f), which supports the conclusion that tissue CD8^+^ T cell expansion may occur primarily through local tissue expansion and not recruitment from other anatomic compartments. In contrast, the abundance of resting and cycling CD4 T cells was not significantly different in MC compared to controls (Fig. 2d, f). Expression of genes associated with circulating T cells (*SELL*, *S1PR1*, *KLF2*)^38,39^ was absent in all expanded CD8 T cell populations (Supplementary Fig. S13); the only cluster with notable expression of circulatory genes was the naïve cluster, which was not expanded in MC. A recent report examining irColitis showed that a subset of tissue *EOMES*^Hi^ CD8 T cells expressed circulating markers and had increased TCR sharing with blood CD8 T cells^15^. In MC, tissue *EOMES*^Hi^ CD8 T cells were notably not expanded in patients with MC compared to controls (Supplementary Fig. S13).

**Fig. 4:**
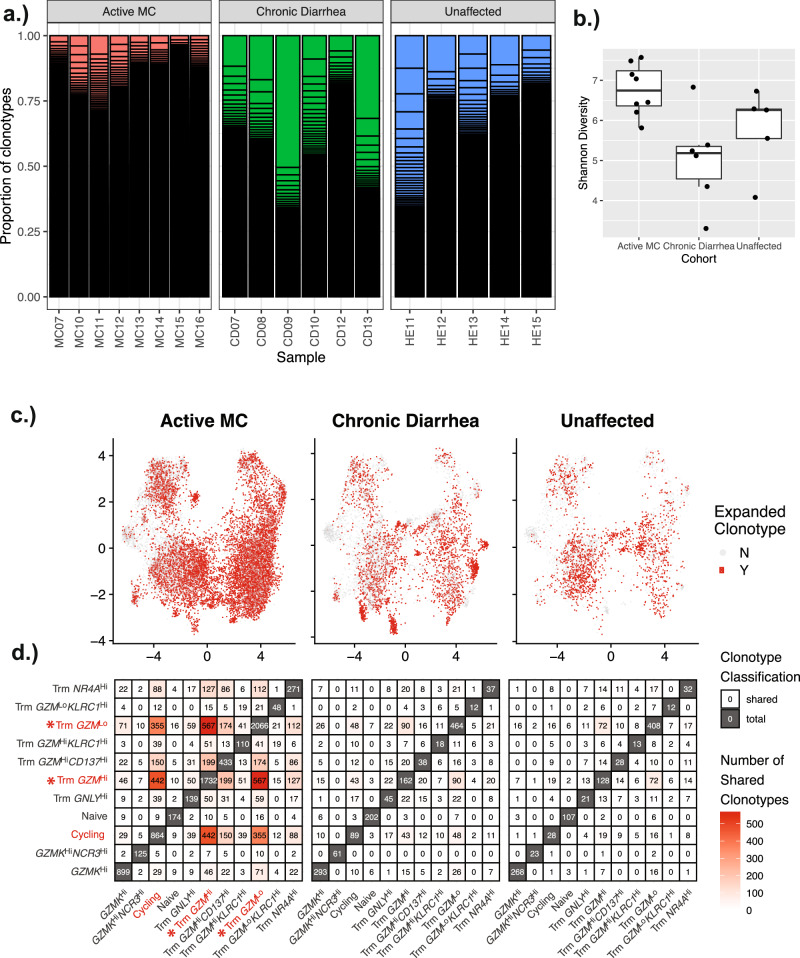
TCR Clonotype analysis identifies increased TCR diversity and points to local replication of CD8 T cells driving the enrichment of *Gzm*^Hi^ Trm cells. **a** TCR clonotypes were merged and a clonotype frequency table was constructed for each patient. The composition was plotted as a stacked bar plot. Each bar represents a unique clonotype, and the height of the bar is scaled based on the proportion. **b** Shannon diversity of the clonotypes was calculated for each patient, and plotted as a boxplot. Number of patients in each cohort: MC (*n* = 8); chronic diarrhea (*n* = 6); unaffected controls (*n* = 5). Boxplot center line represents the median; the box bounds span from the first to the third quartile; whiskers extend from the box to the largest value no further than 1.5 * inter-quartile-range from the box. **c** Clonotypes were classified as expanded if they are present in at least two cells. UMAP plot for CD8 T cells is shown, separated by cohort. Cells with an expanded clonotype are marked in red. **d** Cross-cluster clonotype sharing is visualized as a heatmap. Each cell in the plot illustrates the number of clonotypes shared between the cluster on the *x*-axis and the cluster on the *y*-axis. The cells on the diagonal represent the total number of unique clonotypes in the corresponding cluster. Clusters labeled in red were identified in the scRNAseq compositional analysis as potentially consequential for disease pathogenesis, and clusters demarcated with * exhibit significantly different clonotype sharing between MC and controls when analyzed using a two-sided Fisher’s Exact Test. Number of patients in each cohort: MC (*n* = 8); chronic diarrhea (*n* = 6); unaffected controls (*n* = 5). Source data are provided as a Source Data file.

To better understand the clonal relationship between expanded tissue T cells in MC, single-cell antigen receptor sequencing was performed on tissue T cells using the 10X Chromium platform (*n* = 8 MC, 6 CD, and 5 unaffected controls). The majority of expanded TCR clonotypes were restricted to the CD8 T cell compartment. CD8 TCR clonotypes in MC were more diverse than those from controls (Fig. 4a, b). Although the top clonotypes in controls exhibited a greater per-clonotype expansion than in MC (corresponding with a decreased diversity), MC is characterized by a broad and more uniform expansion of most detected clonotypes (Fig. 4c). Next, TCR clonotype sharing between clusters was examined to better understand the ontologic relationship between tissue CD8 T cell subpopulations. Interestingly, a high degree of sharing between CD8 Trm *GZM*^Lo^, *GZM*^Hi^, and Cycling clusters was observed in MC, with *GZM*^Lo^ and *GZM*^Hi^ Trm cells presenting with significantly different shared clonotype ratios in MC as compared to controls (Fig. 4d). Together these results point to local expansion and not cell recruitment or differentiation as the driving cause of cytotoxic CD8 T cell expansion in MC.

### MC patients have increased colon mucosal regulatory T cells and Th1 cells compared to controls

**Fig. 5:**
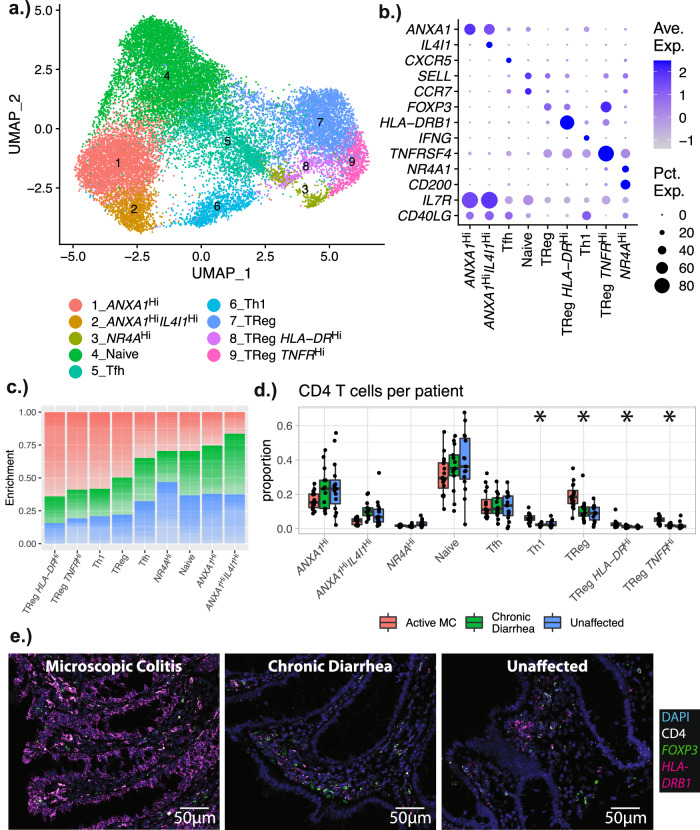
Subclustering of CD4 T Cells. **a** UMAP plot showing the subclustering of CD4 T cells, and corresponding cluster designations. **b** Dot plots showing the distribution of representative markers for the identified cell types. **c** Stacked bar plot demonstrating the relative enrichment of each CD4 T cell subset by cohort designation. **d** Boxplots showing the per-patient proportional differences from each of the subtypes. The proportions shown are relative to the total number of CD4 T cells for each patient. Significance was calculated using scCODA—n.s. indicates the MC proportions are not significantly different from controls, and an asterisk (*) indicates significance at an FDR level of 0.05. Number of patients in each cohort: MC (*n* = 16); chronic diarrhea (*n* = 13); unaffected controls(*n* = 15). Patients with no cells in a given identity class were not plotted. Boxplot center line represents the median; the box bounds span from the first to the third quartile; whiskers extend from the box to the largest value no further than 1.5 * inter-quartile-range from the box. **e** RNAscope images of a representative patient from each cohort. Number of slides examined for each cohort: *n* = 8 for active MC, *n* = 8 for CD, *n* = 9 for unaffected controls. The fluorescent images are colored as follows: DAPI: blue; CD4: white; *FOXP3*: green; *HLA-DRB1*: magenta. Quantitation of all patient slides and individual channels is shown separately in Supplementary Fig. S12. Source data are provided as a Source Data file.

Subclustering of CD4 T cells revealed populations with a naïve phenotype, follicular helper T cells, Th1 cells, cells expressing *ANXA1*, cells expressing *NR4A* transcription factors, and multiple subsets of *FOXP3*^Hi^ T regulatory (Treg) cells (Fig. 5a, b). Compared to controls, patients with MC showed tissue expansion of the Th1 and all Treg populations (*FOXP3*^Hi^ *HLA-DR*^Lo^ *TNFR*^*Lo*^ and two populations enriched for either *HLA-DR* or *TNF* family receptors) (Fig. 5c, d). All Treg and Th1 populations exhibited the same trends in both 10X Genomics and inDrops datasets (Supplementary Fig. S6E).

IHC and RNAscope^37^ were used to characterize the tissue localization of the Tregs expressing CD4 and FOXP3 (*FOXP3*). CD4^+^ T cells were mostly absent from the epithelial compartments and localized to the lamina propria. Within the lamina propria, both total CD4^+^ and FOXP3^+^ (*FOXP3*^Hi^) cells were significantly more abundant in MC than controls. (Fig. 5e, Supplementary Figs. S12, S14, S15).

### MC is defined by the tissue expansion of neutrophils, activated dendritic cells, and *MMP12*^Hi^ macrophages

Although the frequency of myeloid cells did not change across patient cohorts (Fig. 2), there is qualitative evidence of myeloid subcluster remodeling in MC (Supplementary Fig. S3). Compared to controls, patients with MC showed increased tissue neutrophils (*n* = 83 across 27 patients) and activated dendritic cells (*LAMP3*^Hi^, *CCR7*^Hi^, *CD83*^Hi^, *FSCN1*^Hi^, *MARCKSL1*^Hi^; *n* = 120 across 28 patients). These subpopulations represented rare cell types in our dataset, which was consequently underpowered to detected statistically significant abundance changes between patient cohorts. However, the signal was consistent across multiple individuals, which likely reflects the reproducibility of these trends. Activated dendritic cells in MC expressed high levels of proinflammatory *CXCL9* and *CXCL17* chemokines compared to both control cohorts (Supplementary Fig. S16), but these genes did not reach significance. Most of the data for neutrophils were derived from inDrops libraries, as 10X sequencing technology does not readily capture neutrophils^40^ (Supplementary Fig. S6C). Clinical studies report elevated fecal calprotectin, an ion-binding acute phase protein primarily found in neutrophils, in some patients with MC^41,42^, which is consistent with the expanded tissue neutrophils observed in these scRNAseq data.

Macrophages (*n* = 1250 cells across 41 patients) did not show global changes in MC compared to controls. However, abundance shifts were observed within macrophage subclusters that were defined by the differential expression of *CCL4*, *KLF6*, *MMP12*, and metallothionein genes (Supplementary Fig. S17). *MMP12*^Hi^ macrophages exhibited the strongest enrichment, while metallothionein gene-expressing macrophages showed the strongest depletion in MC compared to controls. *MMP12* expression has been linked to pro-inflammatory macrophages^43^, while metallothionein genes *MT1* and *MT2* are upregulated after stimulation with pro-inflammatory cytokines^44^. These data suggest that myeloid compartment remodeling may contribute to MC pathogenesis.

### CD8 T cells exhibit signs of T cell receptor engagement and express both proinflammatory cytokines and *IL10*

**Fig. 6:**
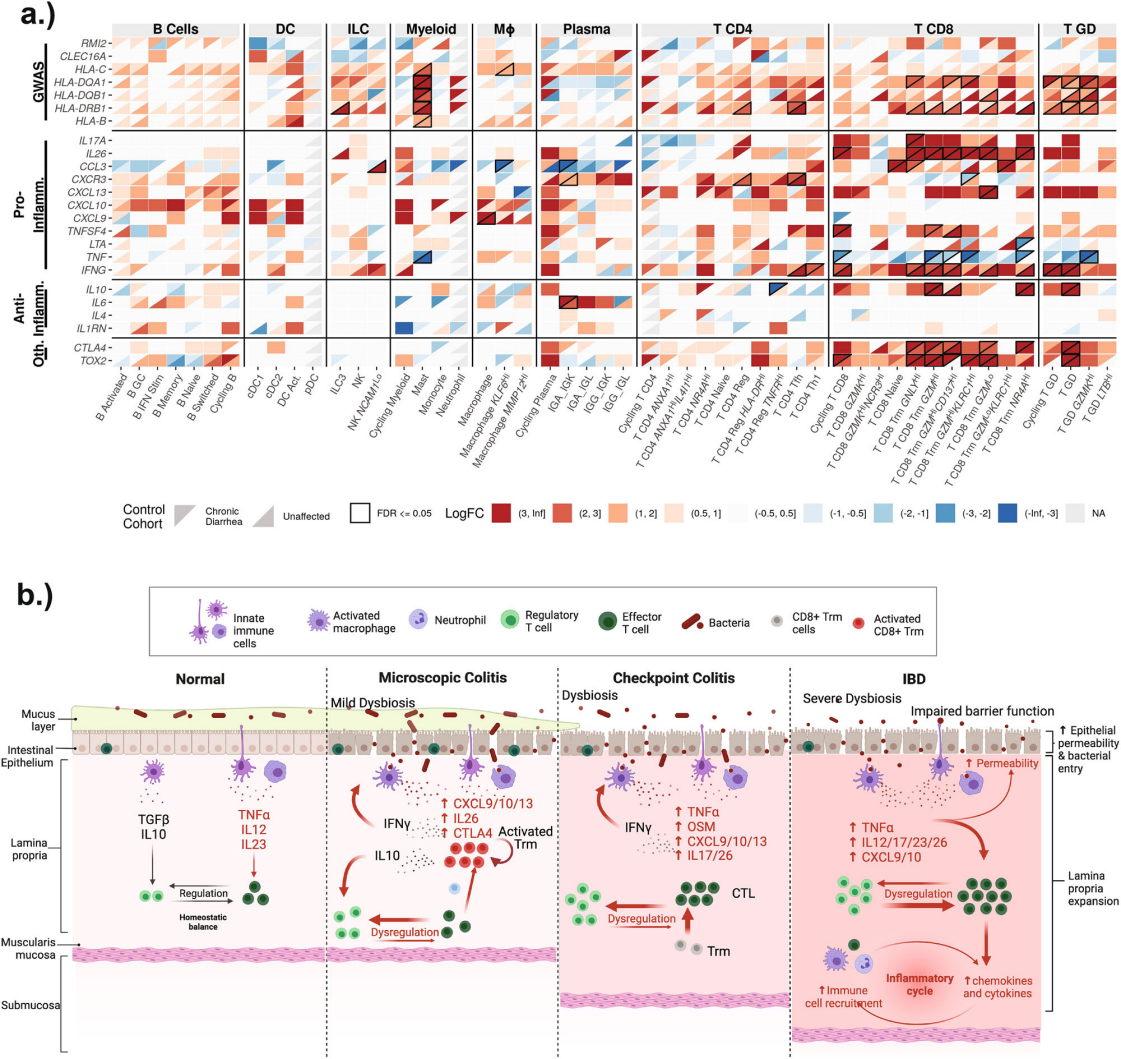
A comprehensive pathologic model of colitides. **a** Pseudobulk analysis was used to compare MC expression profiles to both chronic diarrhea and unaffected control cohorts. A selection of informative genes is shown here, with more shown in the supplement (Supplementary Fig. S16). Expression comparisons are plotted as a heatmap, with rows representing genes, and columns representing cell types. Color indicates level of fold-change; the top-left of each box represents MC vs. chronic diarrhea fold-change, while the bottom-right of each box represents MC vs. unaffected controls fold-change. Comparisons that reached statistical significance (FDR < 0.05) are bounded by a black border. Genes are grouped based on their classification: GWAS (genes previously implicated in GWAS studies); Pro-Inflamm. (a selection of pro-inflammatory genes); Anti-Inflamm. (a selection of anti-inflammatory genes); Oth. (other genes that are indicative of TCR engagement). **b** A model summarizing our findings, in the context of existing literature for IBD and checkpoint inhibitor-induced colitis (irColitis), is shown. Microscopic Colitis, characterized by a relatively mild dysbiosis and no loss of mucosal integrity, is contrasted with irColitis and IBD, where dysbiosis, epithelial damage, and loss of mucosal integrity are common. Elevated *CXCL9/10* and *IL26* are a common theme among colitides. Elevated *INFG*, produced by CD8 T cells, is also common to all colitides. MC has an over-expression of anti-inflammatory IL10, and downregulation of TNF. Dysregulation of the CD4 Treg compartment is a common theme among colitides, presenting in MC as an expansion of CD4 regulatory cells. MC patients are further characterized by a shift in the CD8 Trm T cell compartment towards cells with an activated phenotype. Created in BioRender. Halvorsen, S. (2025) https://BioRender.com/b13b957.

Next, the expression of pro-inflammatory and regulatory cytokines was examined between MC cases and controls. MC cases showed upregulation of proinflammatory cytokines in CD8 T cells, with the strongest signal coming from *IFNG*. Interestingly, *TNF* was mostly downregulated in CD8 T cells in MC cases, although the TNF superfamily member *TNFSF4* was upregulated (Fig. 6a, Supplementary Fig. S16). Furthermore, tissue CD8 T cells had enriched expression of the regulatory cytokines *IL10* (a well-characterized anti-inflammatory cytokine in the gut^45,46^) and *IL26* (an IL10 family cytokine that might play a protective role in inflammatory colitis^18,47–49^) (Fig. 6a, Supplementary Fig. S16). Genes associated with TCR engagement, including *TOX2* and *NR4A1/2* were strongly upregulated across CD8 T cells in MC, indicating that cell expansion likely occurs through TCR engagement and not bystander proliferation.

### Receptor-Ligand analysis identifies potential crosstalk between T and myeloid cells in MC

CellPhoneDB^50^ was used to identify putative signaling networks between cell types in the scRNA-seq dataset. Interactions were considered across all tissue cell types (epithelial, immune, and stromal), and minimal cross-talk between the three major cell lineages was observed (Supplementary Fig. S18A). However, two potential MC-associated cell-cell interactions were observed: *CXCL11*^Hi^ enterocytes interacting with *CXCR3*^Hi^ T cells, and *CD226*^Hi^ T cells binding to *NECTIN2*^Hi^ enterocytes (Supplementary Fig. S18B). CD226 is an immunoglobulin superfamily member that may participate in autoimmunity by directing the migration and activation of CD8^+^ T cells^51^, and the NECTIN-CD226 pathway may shape IEL activation^52^.

Within the immune compartment, CellPhoneDB identified many potentially significant interactions (Supplementary Figs. S19, 20). *CXCL9, CXCL10*, and *CXCL13* are notable chemokines with differential signaling in MC as compared to unaffected controls (receptor-ligand interactions are significant in MC but not controls, and individual genes are upregulated in MC compared to controls) (Fig. 6a, Supplementary Fig. S16, S20). In the current dataset, MC-associated enrichment of *CXCL13* is observed predominantly in T cells, while *CXCL9* and *CXCL10* enrichment is seen in macrophages (Fig. 6a). The CXCR3 chemokine ligands *CXCL9, CXCL10*, and *CXCL11* are interferon-induced genes (ISGs) that have broad roles in T cell chemotaxis and polarization^53^.

To better understand the impact of cytotoxic gene programs upregulated in MC, pro-inflammatory cytokines were examined for differential expression in MC. *IFNG* exhibited the strongest and most consistent upregulation in MC (Supplementary Figs. S16, S20). Increased *IFNG* was detected broadly across all tissue CD8 T cells, especially activated *NR4A*^Hi^ and *GZM*^Hi^ T Cells (Fig. 6a). Numerous myeloid cells express the genes encoding the IFN-G receptor (*IFNGR1, IFNGR2*), including subsets of macrophages and monocytes (Supplementary Fig. S20). Many myeloid cells also upregulated ISGs in MC as compared to controls (Supplementary Fig. S21), providing further evidence of T-cell and myeloid crosstalk in the colon mucosa of patients with MC.

### GWAS-associated genes are enriched in T cells and mast cells

GWAS studies have uncovered a significant association between multiple genes, including specific HLA alleles, and MC. To better understand the cell-type gene expression patterns of these GWAS-identified genes, their expression changes were charted across the diverse cell populations in the scRNA-seq data. Multiple studies have shown that the MHC-II gene *HLA-DRB1* has the strongest genetic association with MC^54–56^, while *CLEC16A* and *RMI2* were only found to have marginal associations with MC^56^. Unsurprisingly, the canonical antigen presenting cells expressed high levels of MHC-II, including *HLA-DRB1* (Supplementary Fig. S22). When examining the GWAS-identified genes for expression changes across cohorts, the strongest MC-associated signal was in *HLA-DRB1*. The increased expression of *HLA-DRB1* was ubiquitous across the CD8 and gamma-delta T cell compartment (Fig. 6a). CD4 T cells in MC patients also upregulated *HLA-DRB1*, but the increase was significant only in Treg and T follicular helper cells. Mast cells were the only other non-T immune cell type with increased expression of the GWAS-implicated HLA genes (Fig. 6a). In RNAscope data, *HLA-DRB1* was also found strongly upregulated by epithelial cells in patients with MC. No significant MC-associated changes in *CLEC16A* or *RMI2* expression were observed.

## Discussion

Here, we presented a comprehensive single-cell analysis of MC and identified significant remodeling of the T cell and myeloid compartments in the disease state. Previous studies of MC have relied on IHC or flow cytometry to identify tissue-expanded lymphocytes as primarily CD8^+^ T cells. We confirmed that most MC-enriched lymphocytes are CD8 T cells and detailed the phenotypic heterogeneity among these expanded populations. Specifically, we observed that a subset of CD8 Trm T cells with an activated cytotoxic phenotype were associated with MC. Interestingly, we did not observe enrichment of *GZMK*^Hi^ CD8 T cells (which have previously been shown to be aging-associated cytotoxic effector cells^57,58^) in MC, a condition that almost exclusively affects older adults^59^. We also provided additional data that support local activation and expansion of CD8 T cells in MC, consistent with prior literature^13,60,61^. Notably, evidence of TCR engagement coupled with elevated TCR diversity suggests that a detailed examination of antigenic specificity in the expanded CD8^+^ T cells could provide important insight on drivers of local T cell activation in MC.

*HLA-DRB1 is* one of the genes with strong enrichment in MC CD8 T cells, and was implicated in previous GWAS studies^54–56^. Histologic validation showed a strikingly high *HLA-DRB1* expression in the epithelial layer. HLA-DRB1 is an MHC class II molecule and is normally only expressed on professional antigen-presenting cells (APCs), but can be induced in non-APCs by interferon-gamma^62^. The observed pan-epithelial increase of *HLA-DRB1* in MC is, therefore, likely mediated by interferons, including those produced by cytotoxic CD8 T cells. Prior studies have shown some intestinal stem cells constitutively express MHC-II, and these cells can communicate with Th cells to affect cytokine production and stem cell differentiation^63^. The established epithelial MHC-II:Th cell axis raises the possibility that the epithelial expression of *HLA-DRB1* could mediate some of the inflammatory cytokine production in MC. However, the lack of CD4^+^ helper T cell infiltration in the epithelial layer may limit the magnitude of this effect.

We identified and validated two MC-associated genes, *LINC02446* and *BATF*. *LINC02446* is a long non-coding RNA previously associated with CD8^+^ memory T cells^64^ and is correlated with better patient survival in bladder cancer^65,66^. *BATF* is a transcription factor involved in CD8^+^ T cell differentiation; *BATF* is induced upon stimulation, enhances/prolongs the effector T cell response, and encourages the development of memory T cells^67,68^. The increased expression of *LINC02446* and *BATF* in MC collectively pointed to sustained stimulation of CD8^+^ T cells and the development of memory cells that could explain the high rate of MC recurrence following discontinuation of initial steroid therapy^69^.

Recent scRNAseq studies of UC and irColitis^14–16^ have provided an opportunity for a global view of inflammatory colitides (Fig. 6b). Although all colitides present with an expansion of the CD8 T cell compartment, the increase in MC appears to be driven predominantly by local expansion in response to TCR engagement. In contrast, the CD8 T cell increase in UC and irColitis have a significant contribution from circulating T cell recruitment^14–16^. Although *IL10* was expressed by a relatively small proportion of cells (0.9% of MC CD8 T cells), it was significantly over-expressed in multiple types of CD8 T cells in MC. *IL10* is an immunosuppressive cytokine that has not been reported to play a major role in irColitis^14,15^. However, polymorphisms in the *IL10* locus (proposed to result in decreased expression^70^) are associated with an increased risk of inflammatory bowel disease (IBD)^71,72^, and defective *IL10* expression or antibodies against it are observed in a subset of patients with IBD^73,74^. Prior attempts at interrogating expression levels of IL10 in UC have yielded mixed results, with some studies reporting an increase^75–77^ and others a decrease or no change^78–80^. Given the well-documented anti-inflammatory properties of IL10^81–85^, the *IL10* increase observed here suggests MC might have a partially functional negative feedback loop limiting the extent of inflammation and epithelial damage.

This study also points to several potential therapeutic opportunities for MC. Given the central role of INF-G, corticosteroids are expected to effectively treat this disease, as demonstrated in recent clinical trials^69,86–89^. Aside from *TNF*, the inflammatory chemokine and cytokine signature in MC was remarkably similar to both irColits and UC, suggesting that therapies effective against UC could also work for MC. All three colitides shared an upregulation of *IL26*, *CXCR3*, and *CXCL13* in the T cells, and an enrichment of *CXCL9, CXCL10*, and *CXL11* in myeloid cells. These common threads highlight the importance of IL-26 and the CXCR3-CXCL9/CXCL10/CXCL11 signaling pathways. Anti-IL-26 therapy has been proposed for other inflammatory diseases, such as psoriasis and IBD^90^. However, the role of IL-26 in this context is poorly understood, with studies supporting both a proinflammatory^47,48^ and a protective role^18^. IL-26 binds the IL-20R1/IL-10R2 receptor, activating the JAK-STAT pathway^47,91^. IFN-G also signals via the JAK-STAT pathway^92^, pointing to a potential common mediator for two prominent upregulated MC cytokines. It is difficult to predict the effects of JAK inhibitors due to complicated downstream signaling. However, inhibitors of the JAK-STAT pathway are effective in treating UC, with two FDA-approved agents^93^. These data and two recent case reports suggest that JAK inhibitors could be effective in MC^94,95^.

We acknowledge several limitations of our study. The main analysis did not distinguish between lymphocytic and collagenous colitis subtypes of MC due to the limited number of participants in each group. However, the central signature (cytotoxic phenotype shift in CD8 Trm cells, and enrichment of CD4 Treg cells) remained consistent across the different histologic subtypes (Supplementary Fig. S23). Further, recent studies suggest MC is a continuum between two extreme endpoints^11^, rather than two discrete subtypes. Although the inDrops libraries were generated from unbiased sampling of all cells within each biopsy, limited numbers of stromal and epithelial cells were recovered, precluding comprehensive analysis of these cell types. Both inDrops and 10X libraries were generated with a single-stage enzymatic dissociation protocol, which did not separate IELs from lamina propria lymphocytes (LPLs). There are currently no well-validated transcriptional markers to separate IELs from LPLs, so the scRNAseq data could not conclusively link the full mRNA profile to anatomic localization. However, our histologic validation of a selection of markers provided additional information on tissue localization of relevant immune cells.

This study provides a seminal resource for cellular shifts in MC and helps to place this condition on the spectrum of gut inflammatory diseases. There are currently no FDA-approved treatments for MC. Therefore, these data will help build a much-needed mechanistic model to identify disease-specific biomarkers and therapeutic options. This study identified T cell subtypes responsible for the lymphocytic infiltration in MC and suggested that CD8 T cells likely expand through TCR-driven cycling rather than bystander activation or recruitment from other tissues. Finally, evidence of anti-inflammatory cytokine signaling that might limit epithelial damage was presented. We highlighted similarities and differences between colitides with the hope of improving our understanding of the mechanistic differences across the colitis spectrum.

## Methods

### Cohort assembly and sample collection

The institutional review board at Mass General Brigham and Dana-Farber approved this study (MGB protocols 2015P001333 and 2015P000275, DFCI/HCC protocols 11-181 and 13-416). Biopsies were collected from patients undergoing diagnostic colonoscopy for chronic diarrhea or screening colonoscopy. Informed consent was obtained from all study participants. Study participants were not compensated for their participation. Eight biopsies were taken from the right colon in all patients. This location was selected because prior studies have shown that biopsies from this segment of the colon have the highest yield for diagnosing MC^11^. In addition, this protocol ensured that all biopsies across the three cohorts were taken from the same segment of the colon.

Study participants self-reported sex. Sex and age were not considered as selection criteria for the MC cohort, but were taken into account when assembling the control cohorts to ensure similar distributions across cohorts. The final sample size was 13 for the chronic diarrhea cohort, 16 for the MC cohort, and 17 unaffected controls. The distribution of female sex in the cohorts was 81%, 85% and 56% in MC, chronic diarrhea and unaffected controls, respectively. Similarly, the mean age of participants were 66.8 years, 57.0 years, and 64.5 years for MC, chronic diarrhea and unaffected controls, respectively.

### inDrops library generation, sequencing, and data processing

Biopsies were minced into 2 mm^3^ chunks using a sterile razor in a tissue culture dish containing 5 mL RPMI with 5% FBS and 50 μL Penicillin-Streptomycin (P/S, Thermo Fisher cat. 15140122). Tissue fragments were then placed in a dissociation enzyme mix of 10 mL of RPMI with 2 mg/mL Collagenase A (Roche 10103586001), 0.1 mg/mL DNAse (Roche), and 10 μL P/S. The tissue was dissociated for 15 min at 37C under rotation at a 45-degree angle. The supernatant containing dissociated cells was removed, leaving behind solid settled chunks, and set on ice. A fresh dissociation enzyme mix was added to the remaining tissue fragments, and a second 15-min dissociation was completed. Supernatants from both rounds of dissociation were pooled and cells were pelleted at 400*g* for 5 min at 4C in a swinging bucket centrifuge. Cells were washed with PBS and filtered through a 70 μm cell strainer. Cells were pelleted again and resuspended in 500 μL of PBS supplemented with 0.1% BSA and stored on ice until used. The maximum time between dissociation and cell encapsulation was approximately 4 h.

inDrops libraries were generated by the Harvard Longwood Single-Cell Core. Cells were provided to the core in PBS with 0.1% BSA. Cells were run through a microfluidic chip with barcoded hydrogels, and the inDrops v3 protocol^17^ was applied to generate sequencing libraries with a target of 2000 cells per library.

inDrops libraries were sequenced by the MGH NextGen Sequencing Core on an Illumina HiSeq2500, in either High Output or Rapid mode, depending on the number of multiplexed libraries. Read lengths were R1:65, R2:8, R3:8, and R4:26. All libraries were sequenced to a target depth of 100,000 reads per cell. Basecalling was performed by Illumina’s bcl2fastq software, but no demultiplexing was specified. A custom Python script was used to extract the individual biological library reads from the raw fastq files based on the expected library index provided by the Single-Cell Core facility. Reads were then modified from the inDrops format (data contained in two index reads and two data reads) to a more standardized two-read (R1 and R2) format with R1 containing the cell barcode and UMI, and R2 containing the transcript sequence. Python scripts were used to merge together the data from each biological library, and to generate a list of all possible inDrops barcodes, and this was input to the StarSOLO software. Reads were mapped to the 10X customized GRCh38 reference (version 3.0.0) using StarSOLO (version 2.7.3a), with the following customized parameters: “ --soloType CB_UMI_Simple --soloCBwhitelist {whitelist_file} --soloCBstart 1 --soloCBlen 16 --soloUMIstart 17 --soloUMIlen 6 --soloBarcodeReadLength 0 --soloUMIfiltering MultiGeneUMI --soloCBmatchWLtype 1MM_multi_pseudocounts --soloFeatures Gene --soloStrand Forward --soloUMIdedup 1MM_All --readFilesCommand zcat --soloCellFilter CellRanger2.2 2000 0.99 10”. These parameters were chosen to generate counts as close to the CellRanger pipeline as possible.

### 10X library generation, sequencing, and data processing

Biopsies were cut into pieces less than 1 mm in diameter with dissecting scissors and placed into RPMI media (ThermoFisher 11835055) with 1 mg/mL of protease from *Bacillus lecheniformis* (MilliporeSigma P5380), 5 mM CaCl_2_, and 0.1 mg/ml DNAse I (MilliporeSigma DN25). Tissue dissociation mix was incubated at 4 C for 40 min, under constant rotation. Tissue was triturated every 10 min with a P1000 pipette. The dissociation reaction was stopped by adding human AB serum (MilliporeSigma H4522) to a final volume of 10%. The dissociated mix was filtered through a 70 µM filter to remove clumps, then centrifuged for 10 min at 350 *g*. The cell pellet was resuspended in RPMI media, and cells were counted in a hemocytometer. Cells were then cryopreserved in a 1:1 solution of RPMI: CryoStor CS10 (BioLife 210102) for a final DMSO concentration of 5%.

After thawing a vial of cells, single-cell suspensions were brought up in phenol-free RPMI with 2% (v/v) human AB serum and incubated on ice for 30 min with the following antibodies: CD66b-FITC (1:100, BioLegend, 305104), EpCAM-PE (1:100, BioLegend, 324206), CD45-APC (1:150, BioLegend, 304012), CD3 PerCP-Cy5.5 (1:150, BioLegend, 300328) and CD235a PE-Cy7 (1:150, BioLegend, 349112). Cells were washed once and resuspended in phenol-free RPMI with 2% (v/v) human AB serum containing DAPI (Thermo Fisher Scientific, 62248). Single-color controls were performed with these same antibodies using BD CompBeads (BD Biosciences, 552843). Live (that is, DAPI^−^), singlet, CD66b^−^CD235a^−^EpCAM^−^CD45^+^ cells were sorted into phenol-free RPMI with 2% (v/v) human AB serum. All sorting was performed on Sony SH800 or MA900 cell sorters. At least 20,000 immune cells were sorted from each patient. Isolated CD45^+^ cells were centrifuged and resuspended at a concentration of 800–1200 cells per microliter in RPMI with 2% (v/v) human AB serum and loaded onto a 10X Chromium instrument with the following kit: Chromium Single Cell 5’ V1 (10X Genomics product PN-1000006). The targeted recovery was 4000 cells. The manufacturer’s protocol was followed to generate sequence-ready libraries. TCR amplification/library generation was performed according to the manufacturer’s directions (10X Genomics kit PN-1000005 and PN-1000016). Gene expression libraries were sequenced on an Illumina NextSeq, and the TCR libraries were sequenced on an Illumina MiSeq. 10X Genomics Cell Ranger (version 3.1.0) was used to demultiplex reads, align to the human reference genome GRCh38 (customized by 10X Genomics, version 3.0.0), and generate tables of UMI counts.

### Cell filtering and quality check

The raw, non-filtered UMI (Unique Molecular Identifier) matrices were imported into R (v3.6.2)^96^ using either the Read10X or Read10X_h5 function contained within the Seurat package (v3.1.4)^97^. UMI matrices were then filtered using the EmptyDrops^98^ algorithm with a lower UMI bound setting of 100. Cells were kept if they either had UMI counts above the calculated “knee”, or had a False Discovery Rate (FDR) value below 0.01. The data was further filtered to remove any cells with less than 200 unique genes detected or more than 20% mitochondrial content. This filtering preferentially removed epithelial cells over immune cells, suggesting the epithelial cells were more sensitive to the dissociation conditions. One patient’s library was removed from downstream analysis because the library indicated hallmark signs of cell overloading during the cell encapsulation phase (greater than expected number of cells, with many cells exhibiting multi-lineage markers). One patient with histological signs of MC but no clinical symptoms (asymptomatic MC) was included in the scRNAseq colon atlas but was excluded from any cohort comparisons. Upon subclustering analysis, clusters solely defined by high expression of mitochondrial genes or a lack of marker genes and low numbers of detected genes/UMIs were removed.

### Separating immune, epithelial, and stromal cells

For each library, cells were separated into immune, epithelial, and stromal compartments before any further downstream analysis. The cells were separated based on the approach in a previous report^16^. Briefly, cells were clustered using the standard Seurat pipeline for high-dimensional clustering: data were normalized, variable features were identified, data were scaled, PCA was performed, a shared-nearest-neighbor graph (SNN) was generated, and clusters were identified based on the default SNN modularity optimization algorithm with a resolution parameter of 2. The average expression level (from the “RNA” slot in Seurat) was calculated for each cluster. A cellular compartment score was calculated as the average expression of a set of manually curated genes, and the cluster was named based on the maximum score. The manually-curated genes were taken from a previous report^16^, and are as follows: epithelial – *EPCAM*, *KRT8*, *KRT18*; stromal – *COL1A1*, *COL1A2*, *COL6A1*, *COL6A2*, *VWF*, *PLVAP*, *CDH5*, *S100B*; immune – *CD52*, *CD2*, *CD3D*, *CD3G*, *CD3E*, *CD79A*, *CD79B*, *CD14*, *CD16*, *CD68*, *CD83*, *CSF1R*, *FCER1G*. All cells identified as belonging to each cellular compartment were merged, resulting in three separate datasets for downstream analysis.

### Batch correction and dimensionality reduction

Batch correction was performed on the separated immune, stromal, and epithelial datasets using the Harmony (v1.0) algorithm^99^ and dimensionality reduction was performed via UMAP using Seurat^97^. Briefly, data were normalized, variable features were identified, data were scaled, and PCA was run. Harmony was run for 50 iterations. Harmony was run with two batch variables: technology (10X vs. inDrops) and patient ID. High-dimensional clustering was performed using the Seurat SNN modularity optimization algorithm using the Harmony reduction slot. Finally, UMAP dimensionality reduction was performed with the first 50 dimensions of the Harmony data slot.

### Cluster stability evaluation

Cluster stability was evaluated using the adjusted rand statistic in a bootstrap-like approach, as previously reported^100^. Briefly, data were randomly subsampled without replacement so that 90% of the input data was retained. This was repeated for 20 iterations at each tested resolution parameter. The downsampled data was reclustered with a different random seed for each tested resolution iteration. The adjusted rand index was computed between the original and downsampled datasets by the adj.rand.index function in the fossil package (v0.4.0) for R^101^. The rand indices for each resolution parameter were presented as a boxplot, and the largest resolution before the index started decreasing was chosen for downstream analysis (Examples shown in Supplementary Fig. S24).

### Differential gene expression

Differentially expressed genes (DEGs) were calculated using both a wilcoxon rank-sum test and pseudobulk analysis (see below). For cell type assignment, DEGs were generated comparing one cluster against either all other similar cells (e.g. against all the T cells) or all other cells within the same compartment (e.g. all immune cells). A combination of adjusted *p*-values, average log-fold-change, and percent expression was used to identify top candidates for cell type determination. Cohort-level DEGs (e.g. CD8 Trms from MC patients vs. CD8 Trms from unaffected controls) were used to identify MC-specific transcriptional alterations. DEGs for each cell cluster are included in Supplementary Data 2.

### Pseudobulk analysis

Pseudobulk profiles for each cell group were generated by summing up the raw counts for each gene. Cell groups were specified as the unique combination of cell type and patient ID (e.g. Patient001_CD4-T-Reg, Patient001_CD4-Th1, etc.), and filtered to include only groups with at least 5 cells. Pseudobulk profiles were then processed using either a limma-voom^102^ pipeline to generate lists of DEGs associated with cell type, or an edgeR GLM pipeline^103^ to generate lists of DEGs associated with cohorts within each cell type. Lists of chemokines and cytokines were taken from the Gene Ontology database^104,105^ by searching for “cytokine activity” or “chemokine activity”. The cytokine lists were cross-referenced with proinflammatory (“positive regulation of inflammatory response”) or anti-inflammatory (“negative regulation of inflammatory response”) lists to generate lists of proinflammatory and anti-inflammatory cytokines. Cohort-level pseudobulk DEGs for each cell cluster are included in Supplementary Data 3.

### Compositional analysis

scCODA (v0.1.5)^106^ was used to perform compositional analysis and identify significantly expanded or contracted cell types. Briefly, a table of cell counts was imported into the scCODA python package. MCMC sampling was performed with automatic reference cell type calculation and the covariate set to a binary classifier describing whether the patient has an active MC diagnosis. The significance cutoff value was kept at the default FDR-corrected *p*-value of 0.05. Enrichment plots were generated by first calculating the proportion of a group represented by each cell type (e.g., proportion of MC cells contained in the T CD8 Trm Activated cluster). These proportions were plotted in a stacked column graph, where each column was scaled to 100%, cohorts were colored differently, and varying transparency levels were used to signify individual patient contributions to the cohort-level enrichment.

### Receptor–ligand analysis

Potential signaling interactions were examined using CellPhoneDB^50^ (v5.0.0). Normalized counts were exported from R using the Matrix package^107^. CellPhoneDB was run using the Statistical Analysis Method with default parameters. Interactions were visualized using the ktplots package^50^.

### TCR clonotype analysis

TCR clonotypes were assembled by the cellranger software (10X Genomics). Some patient cells were encapsulated on multiple lanes of a 10X Genomics chromium chip; all identified TCR clonotypes for each patient were merged together based on the assembled CDR3 sequences that cellranger assigned as high confidence and productive (clonotypes from all libraries from one patient were merged and assigned a new unique clonotype ID associated with the unique set of CDR3 sequences). Clonotype frequencies were calculated per patient and plotted as a stacked bar plot. Shannon diversity was calculated for each patient using the vegan package^108^. An expanded clonotype was defined as a clonotype assigned to at least 2 cells. A shared clonotype was defined as an expanded clonotype that is present in at least two distinct cell clusters. To identify clusters exhibiting different clonotype sharing proportions across cohorts, two contingency tables were generated for each cluster. One table compared MC and chronic diarrhea, while the other table compared MC and unaffected controls. The contingency table tabulated the number of shared vs. unique clonotypes within the cell cluster. A Fisher’s Exact Test was performed on the contingency table. The Benjamini-Hochberg procedure^109^ was used to correct for multiple hypothesis testing, and a cluster was considered significant only if the adjusted *p*-value was less than 0.05 in both comparisons (MC vs. chronic diarrhea and MC vs. unaffected controls).

### IHC staining and analysis

IHC staining for CD4, CD8, FOXP3, and GZMB was performed by the MGH clinical immunohistochemistry laboratory. The formalin-fixed biopsy samples were embedded in paraffin, sectioned, and stained with Hematoxylin & Eosin (H&E) following routine protocol. Two pathologists with expertise in gastrointestinal pathology (YK and MM-K) reviewed the H&E-stained slides and confirmed the diagnosis of MC in the study cohort or normal colonic mucosa in the controls. The diagnosis of lymphocytic colitis is based on increased IELs (>20 IELs per 100 epithelial cells) while the diagnosis of collagenous colitis is based on increased IELs and a thickened (>10 μm) subepithelial collagen band. Five random areas per slide were selected for IHC analyses. Positive lymphocytes were counted per high-power field (x400 magnification) of the lamina propria or per 100 enterocytes of the surface or crypt epithelium in the 5 selected areas. A sum of 5 counts for each of the three compartments were recorded in each sample.

Poisson regression was used to compare the number of cells stained across all cohorts. The cell count for each stain was modeled on the predictor variable cohort (MC was selected as the reference value). *p*-Values were adjusted for multiple hypothesis testing using an FDR correction. An adjusted *p*-value cutoff of 0.05 was used for determining significance. A stain was considered significant only if MC was significantly different as compared to both chronic diarrhea and unaffected controls.

### RNAscope staining and analysis

RNAscope staining was performed by the Longwood Neurobiology Imaging Facility. RNAscope Multiplex fluorescent assay V2^37^ (ACD Bio) was performed according to the manufacturer’s directions. In addition to the RNAscope probes, an immunostain for CD4 (ThermoFisher 14-2444-82; dilution 1:100) or CD8 (Leica PA0183; ready to use, no dilution) was used with the secondary antibody Goat anti-mouse (ThermoFisher A-21037; dilution 1:400). Three separate RNAscope panels were used: Panel 1—CD4 (immunostain), *HLA-DRB1*, *FOXP3*; Panel 2—CD8 (immunostain), *HLA-DRB1*, *LINC02446*; Panel 3—CD8 (immunostain), *GZMB*, *BATF*. All slides were scanned using confocal microscopy (Leica Stellaris 5) at 40X magnification.

Images were exported as tiff files from the Leica LasX software. Slides and channels exhibiting gross technical artifacts were removed from further analysis. A region of interest was manually selected from each slide. If possible, regions devoid of technical artifacts that presented crypts, surface epithelial layer, and a significant amount of lamina propria were selected. In most slides, the nuclei in the epithelial layer were too close to automatically separate and generate an accurate cell count. To generate the most accurate count, only the lamina propria was quantitated—a manual mask was drawn using ImageJ^110^ to gate out the epithelial layer. CellProfiler^111^ was used to isolate the manually identified lamina propria layer and export each channel as a separate image for further analysis.

Two quantitation methods were used, depending on the staining patterns. Probes that presented with speckle-like staining (*GZMB*) were quantified using CellProfiler. Probes that stained large areas of the cell (DAPI, CD8, CD4, *HLA-DRB1*, *FOXP3*, *LINC02446*, *BATF*) were quantified using the Ilastik density counting workflow^112^. For the speckle counting workflow, the DAPI channel was first used to segment nuclei. Cell boundaries were inferred from the nuclei based on proximity. Speckles were then enhanced using a feature size of 3. Speckles were then identified using the RobustBackground method. A cell was classified as high for *GZMB* if it contained at least 5 speckles.

To calculate statistical significance, two regression methods were used: Poisson regression (which assumes the variance equals the mean), and quasi-Poisson regression (which tries to correct for over-dispersion by modeling the variance as a linear function of the mean). Both regression methods were performed in R using the glm function. The cell count for each probe was modeled on the predictor variable Cohort (MC was selected as the reference value). The total number of cells in the image (based on the DAPI count) was used as an offset in the regression. A *p*-value cutoff of 0.05 was used for determining significance. The results were largely consistent across both regression methods, with the exception of *BATF* which only reached significance using Poisson regression.

### Reporting summary

Further information on research design is available in the Nature Portfolio Reporting Summary linked to this article.

## Supplementary information

Supplementary Information

Description of Additional Supplementary Files

Supplementary Data 1

Supplementary Data 2

Supplementary Data 3

Reporting Summary

## Source data

Source Data

## Data Availability

The sequencing data generated in this study have been deposited in the dbGaP database under accession code phs003876.v1.p1, which can be found at the following address: http://www.ncbi.nlm.nih.gov/projects/gap/cgi-bin/study.cgi?study_id=phs003876.v1.p1. The sequencing data are available under restricted access as per our IRB protocol. Access can be obtained by submitting a Data Use Certificate to the NIH Data Access Committee for approval. The processed data are available at Zenodo with 10.5281/zenodo.14661972. All other data are available in the article and its Supplementary files or from the corresponding author upon request. Source data are provided with this paper.

## Code availability

Processed data and code for generating the figures is available at Zenodo with 10.5281/zenodo.14661972.
