# Supplementary material for: Single-cell transcriptomic characterization of microscopic colitis
Source: Nat Commun. 2025 May 18;16:4618. doi: 10.1038/s41467-025-59648-8 (PMC12086216; doi:10.1038/s41467-025-59648-8)
Supplement: Supplementary file 1 — Supplementary Information [file 41467_2025_59648_MOESM1_ESM.pdf]

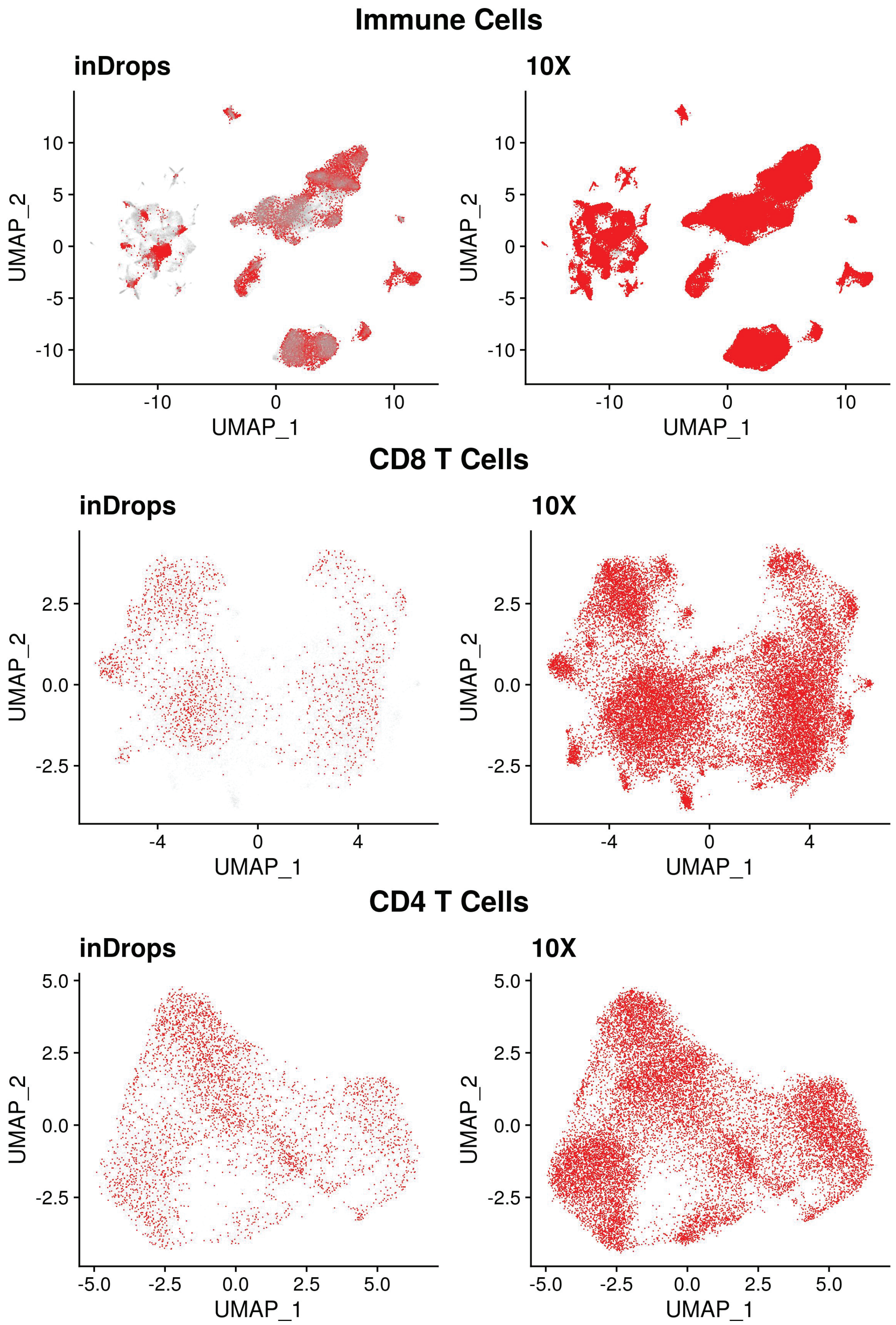

Supplementary Figure 1
UMAP plots illustrating batch correction and clustering. UMAP plots are shown for all immune cells (top), CD8 T cells (middle) and CD4 T cells (bottom). Plots are highlighted to show the cells coming from inDrops libraries (left) or 10X Genomics libraries (right)

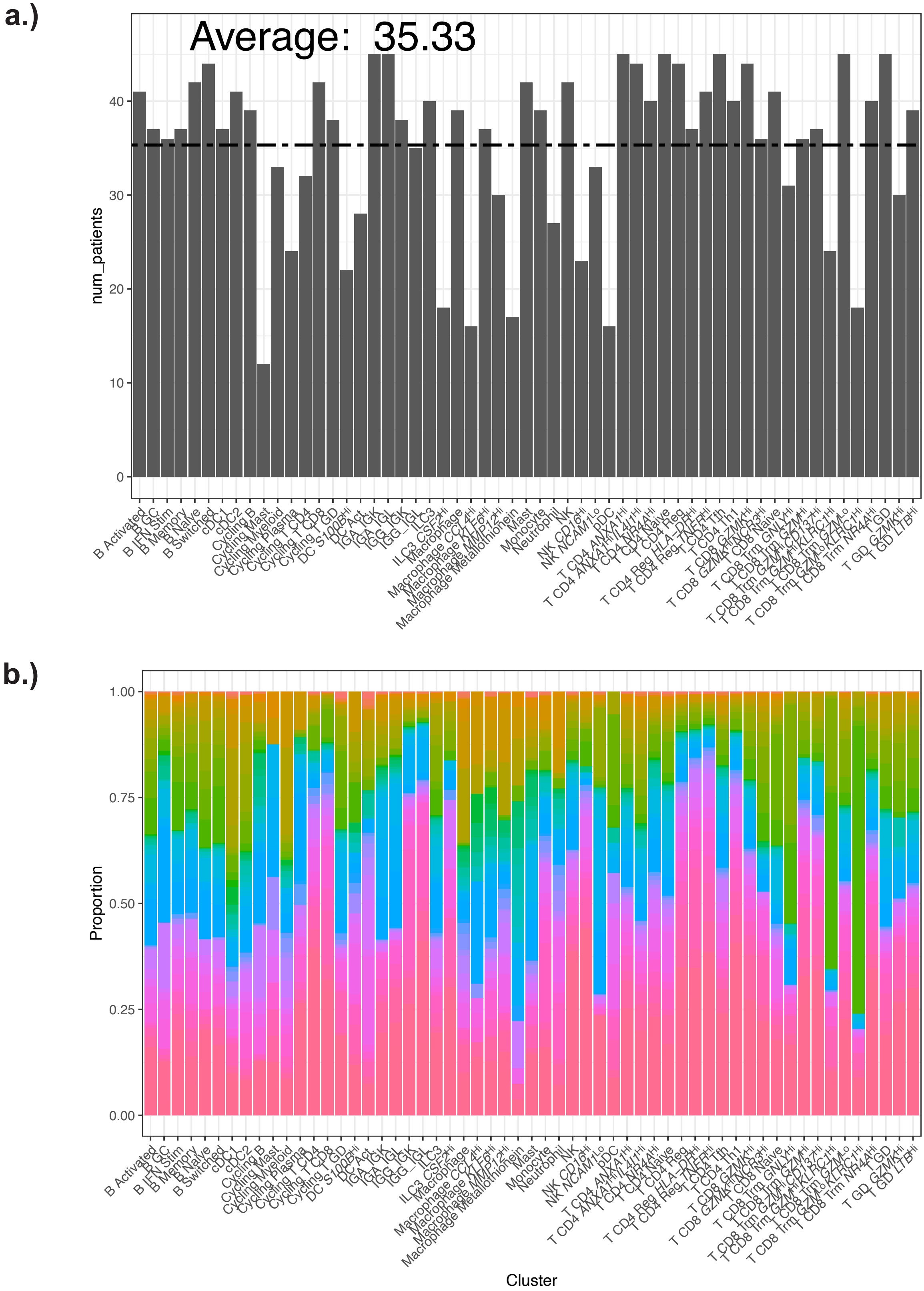

Supplementary Figure 2
(A) Number of patients with cells in each of the recovered cell types. Most cell types are well represented in a majority of patient samples, indicating broad sampling and success of the batch correction procedure. (B) Patient proportions for each cluster. Each patient is represented by a different color. Cell clusters present with composition from a diverse set of patients, indicating broad sampling and lack of patient-driven batch effects.

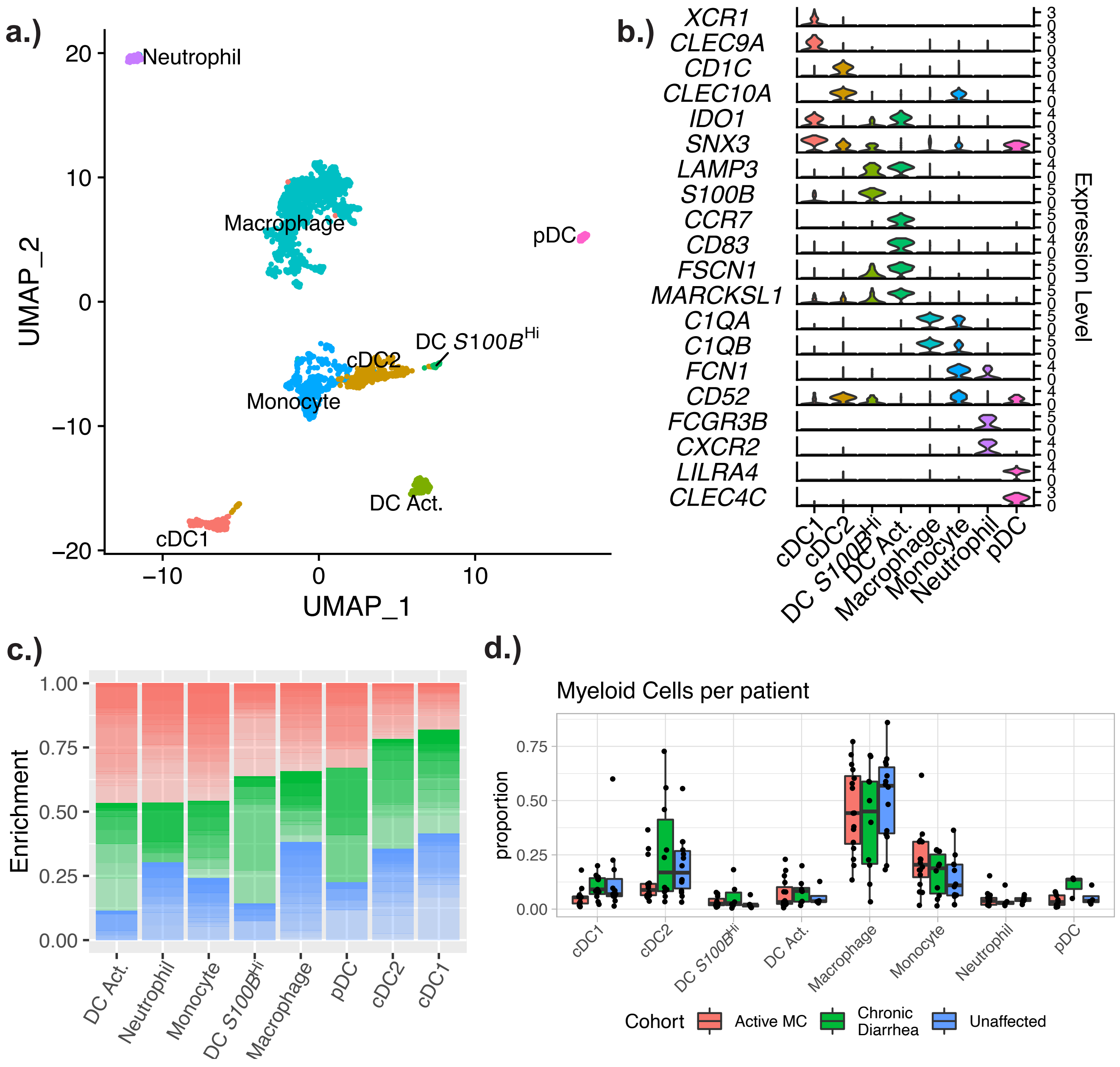

Supplementary Figure 3

A.) UMAP plot showing the subclustering of all myeloid cells, and corresponding cluster designations. B.) Violin plots showing the distribution of representative markers for the identified cell types. C.) Stacked bar plot demonstrating the relative enrichment of each myeloid cell subset by cohort designation. D.) Boxplots showing the distribution of subtypes of myeloid cells. Number of patients in each cohort: MC (n=16); chronic diarrhea (n=13); unaffected controls (n=15). Patients with no cells in a given identity class were not plotted.

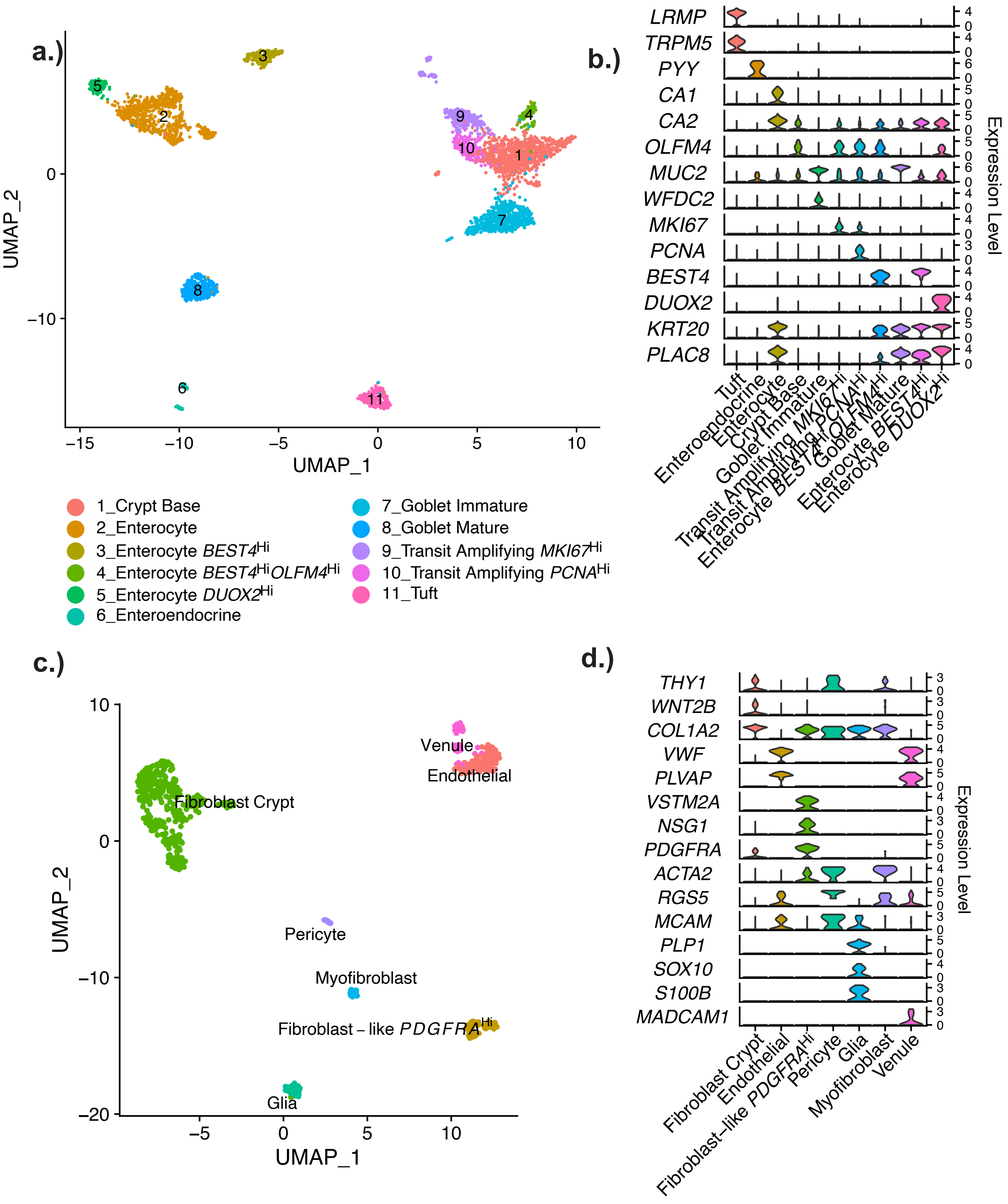

Supplementary Figure 4
UMAP plots of the recovered epithelial cells (A), and recovered stromal cells (C). Violin plots showing expression distribution of representative markers for each epithelial (B) and stromal (D) cell type.

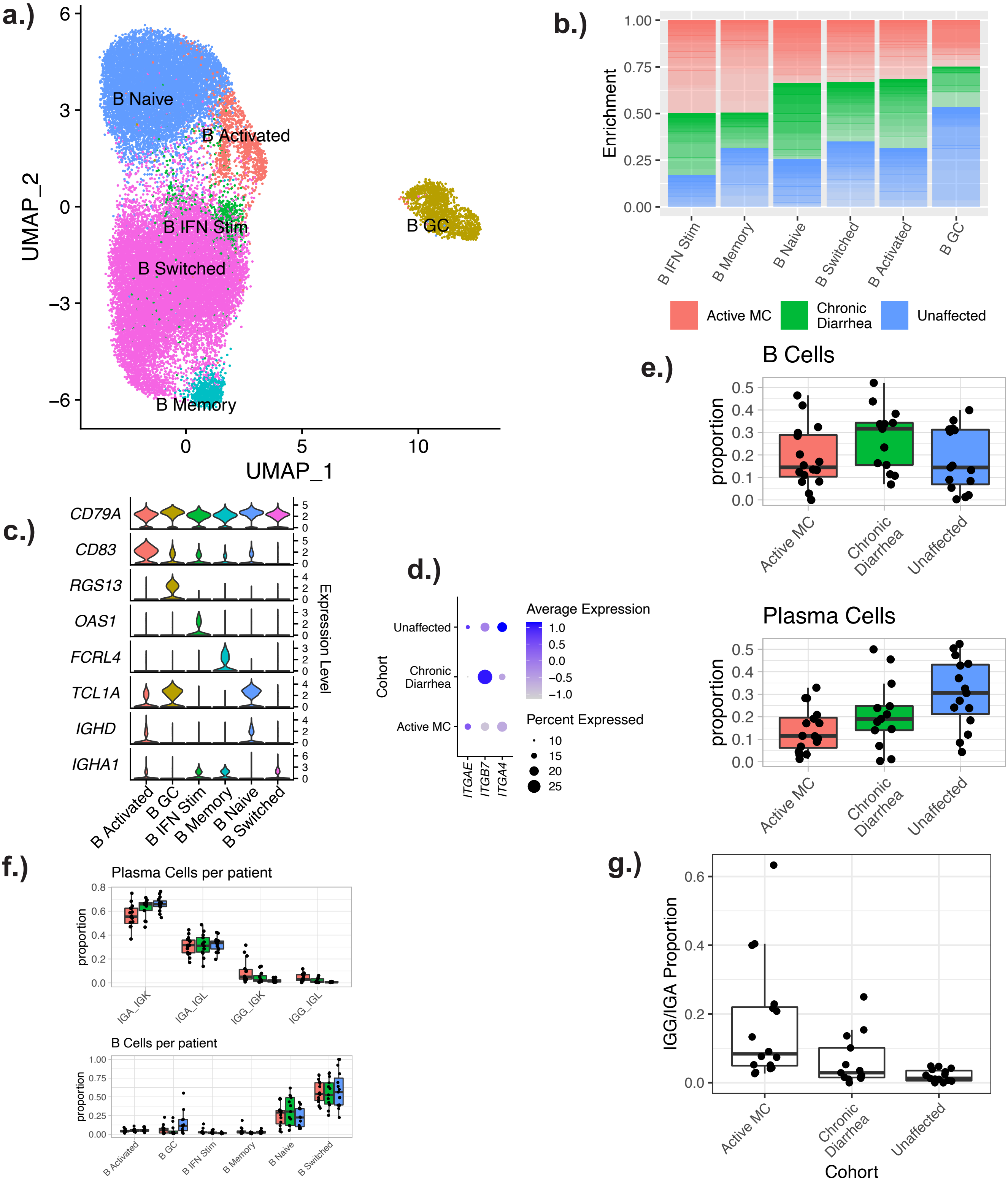

Supplementary Figure 5

A.) UMAP plot showing the subclustering of all B cells, and corresponding cluster designations. B.) Stacked bar plot demonstrating the relative enrichment of each B cell subset by cohort designation. C.) Violin plots showing the distribution of representative markers for the identified cell types. D.) DotPlot illustrating that the decrease in plasma cells for MC patients is likely not caused by an absence of cell trafficking integrins. Plasma cell trafficking integrins are actually marginally increased in MC patients. E.) Boxplots showing the per-patient proportions of B cells and plasma cells. The B cells were not significantly altered in MC patients, while the plasma cells were strongly decreased. Number of patients in each cohort: MC (n=16); chronic diarrhea (n=13); unaffected controls (n=15). F.) Boxplots showing the distribution of subtypes of plasma and B cells. With a formal compositional analysis, none of the B cell subtypes were significantly expanded or contracted in MC patients. G.) The ratio of IGG to IGA plasma cells was calculated for each patient, and plotted as a boxplot (grouped by cohort). Number of patients in each cohort: MC (n=16); chronic diarrhea (n=13); unaffected controls (n=15).

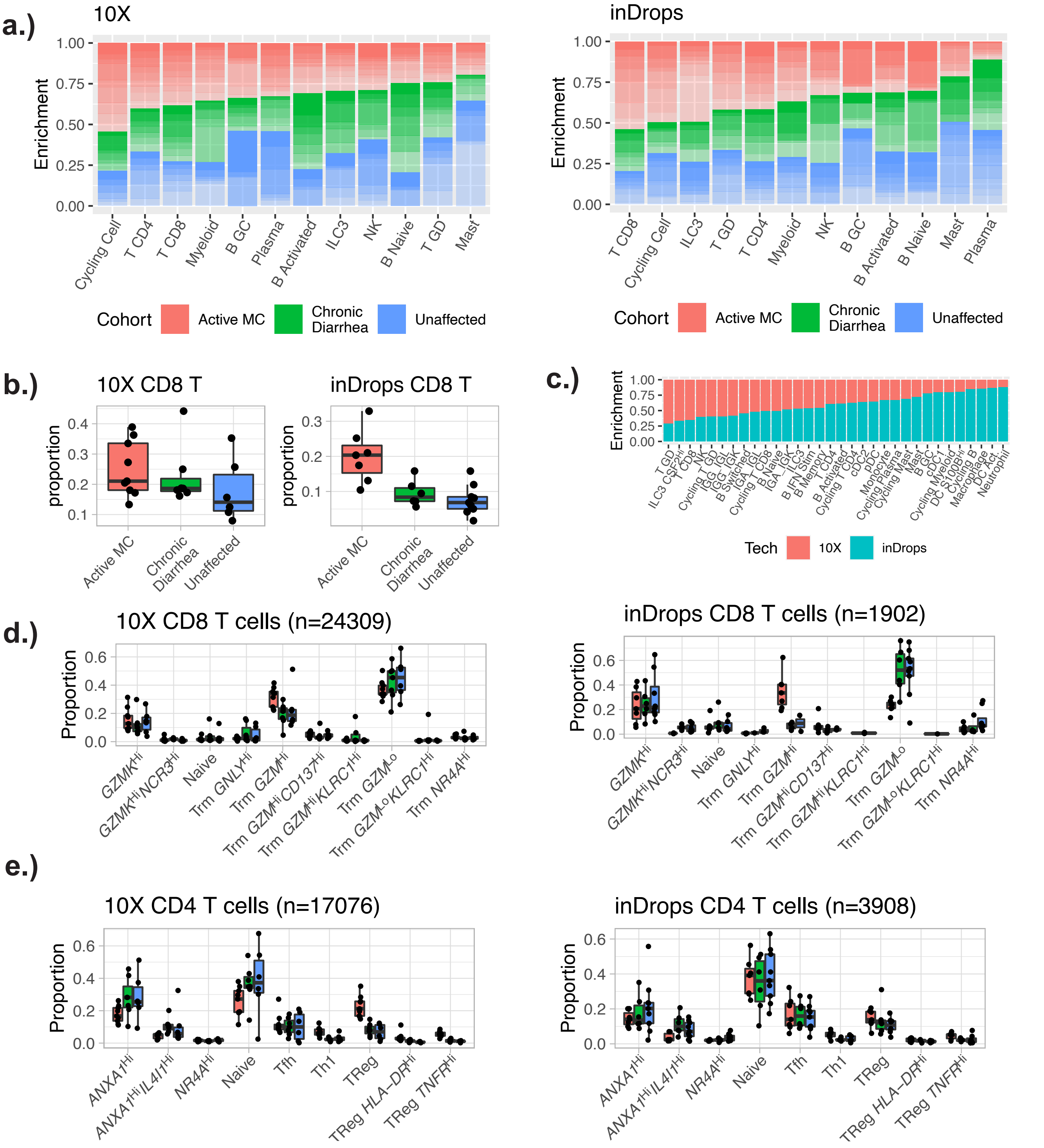

Supplementary Figure 6

A.) Stacked bar plot demonstrating the relative cohort enrichment of each immune cell type, if 10X and inDrops datasets are examined separately. CD8 T cells and cycling cells are increased in MC patients via both encapsulation technologies. B.) Boxplots showing the per-patient proportional differences of CD8 T cells per cohort, segregated by encapsulation technology. Both 10X and inDrops indicate an expansion of CD8 T cells associated with MC. C.) Stacked bar plot illustrating the technology-specific cell capture biases in our dataset. 10X does not capture as many myeloid cells (especially neutrophils) as inDrops, a well documented phenomenon. D.) Boxplots showing the per-patient proportional differences of the CD8 T cell subtypes, grouped by encapsulation technology. The proportions shown are relative to the number of all CD8 T cells for each patient. E.) Boxplots showing the per-patient proportional differences of the CD4T cell subtypes, grouped by encapsulation technology. The proportions shown are relative to the number of all CD4 T cells for each patient. For all panels: Number of patients by cohort in 10X group: MC (n=9); chronic diarrhea (n=7); unaffected controls (n=6). Number of patients by cohort in inDrops group: MC (n=7); chronic diarrhea (n=6); unaffected controls (n=9).

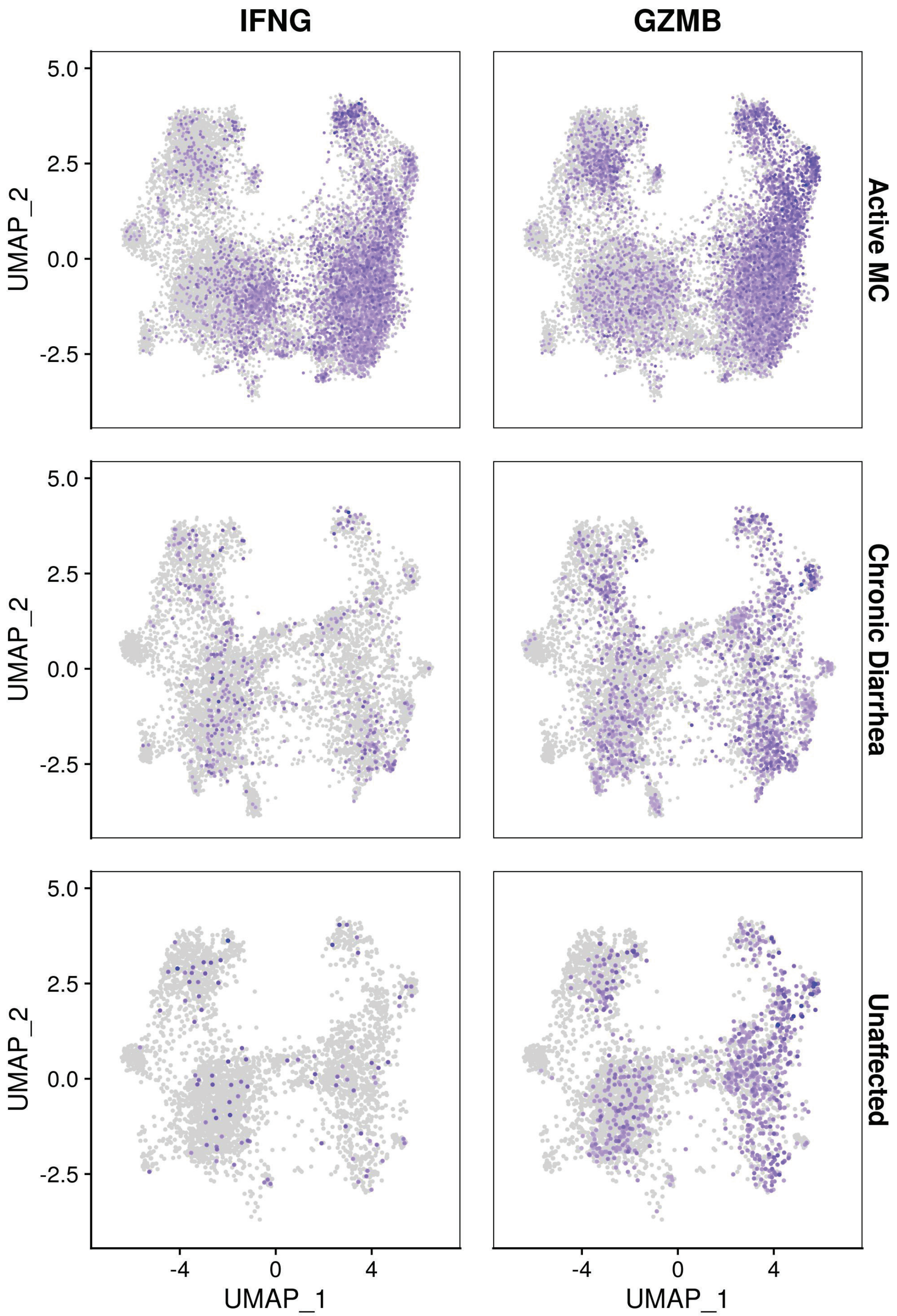

Supplementary Figure 7
Feature Plots illustrating the expression patterns of IFNG (left) or GZMB (right) within the CD8 T cells. Each cohort is plotted on a separate row. The UMAP coordinate for each cell is colored based on the gene expression level (gray = no expression, dark blue = high expression). Most of the cells expressing high levels of IFNG in MC patients also produce high levels of GZMB.

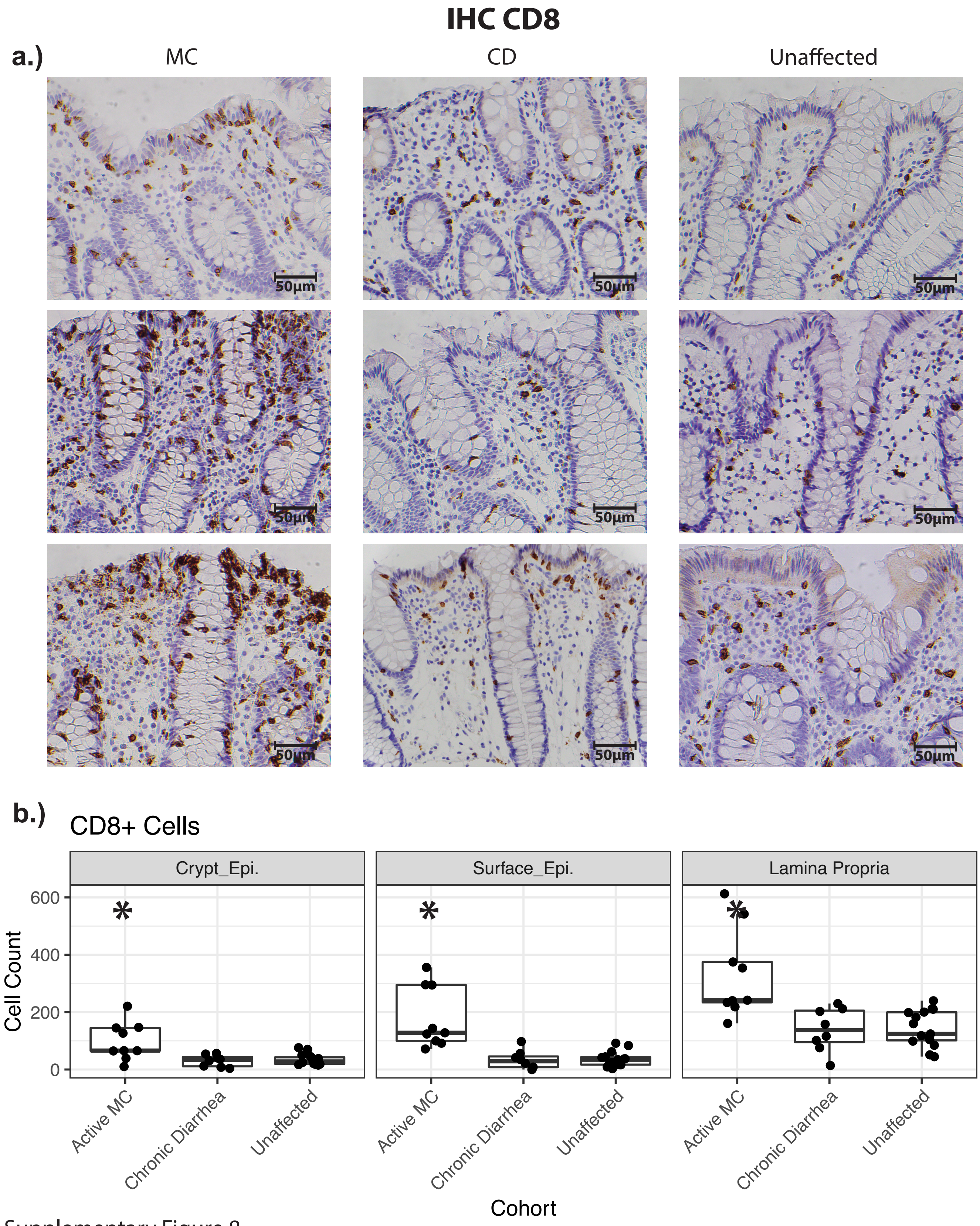

Supplementary Figure 8

A.) Patient biopsies were stained for CD8 using IHC. Representative images from three patients per cohort are shown. B.) Positive cells from all patient slides (n=9 for active MC, n=8 for chronic diarrhea, and n=15 for unaffected controls) were quantitated separately in the crypt epithelial cells, apical epithelial cells, and lamina propria. Cell counts for all slides are plotted as boxplots. An asterisk (*) indicates MC is significantly different from both controls at an FDR level of 0.05.

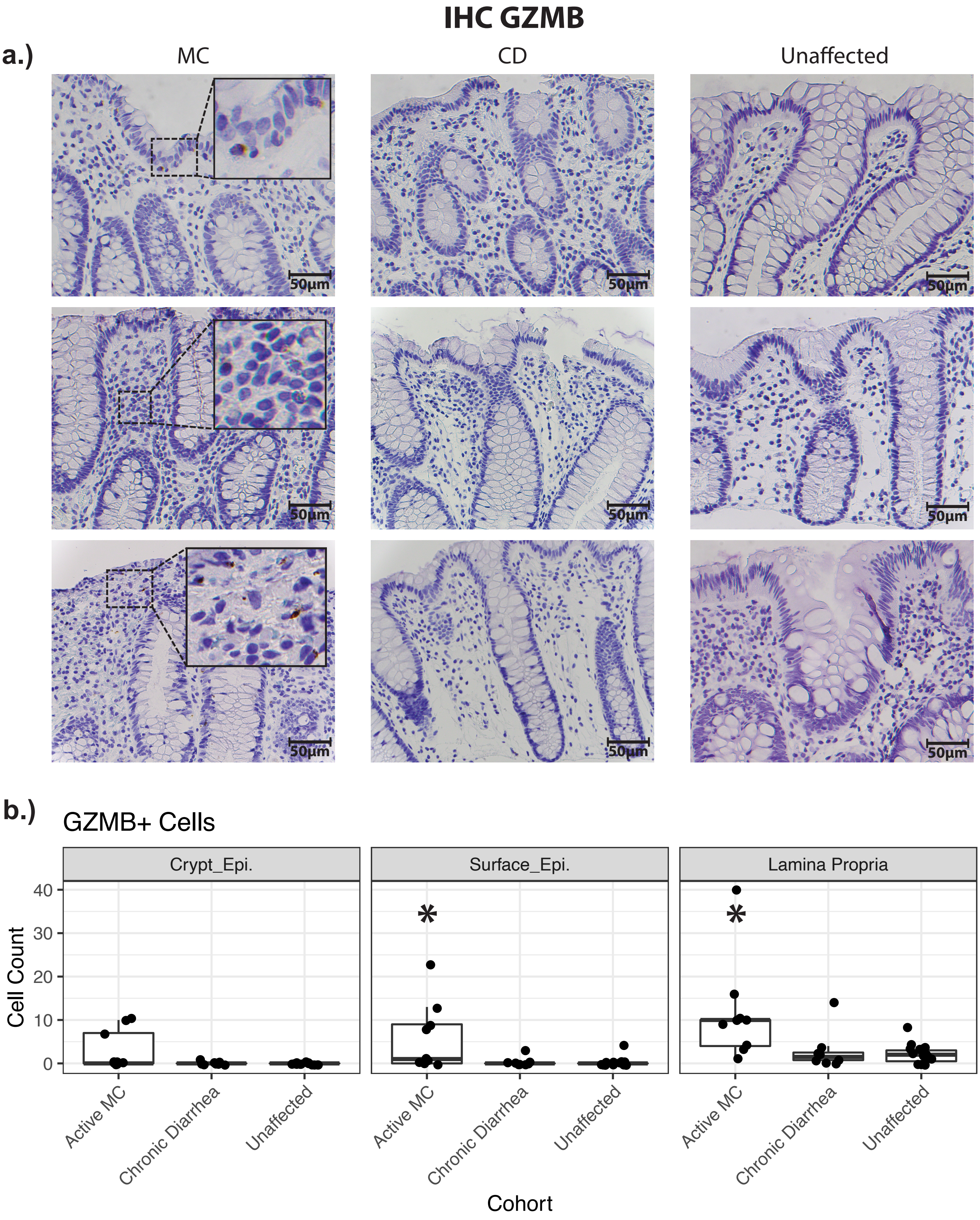

Supplementary Figure 9

A.) Patient biopsies were stained for GZMB using IHC. Representative images from three patients per cohort are shown. B.) Positive cells from all patient slides (n=9 for active MC, n=8 for chronic diarrhea, and n=15 for unaffected controls) were quantitated separately in the crypt epithelial cells, apical epithelial cells, and lamina propria. Cell counts for all slides are plotted as boxplots. An asterisk (*) indicates MC is significantly different from both controls at an FDR level of 0.05.

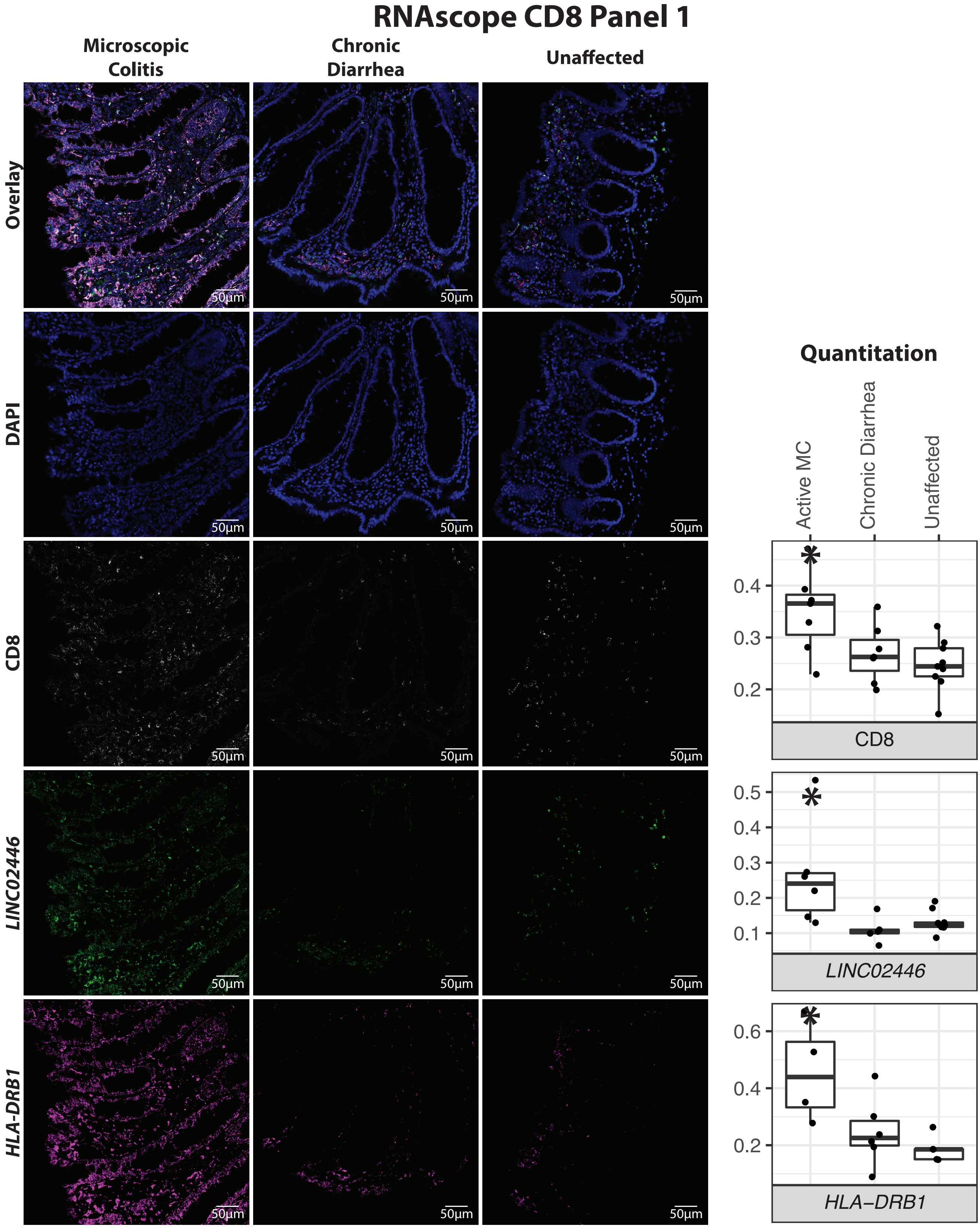

Supplementary Figure 10

A.) Patient biopsies were stained using RNAscope technology and imaged with confocal microscopy. An antibody against CD8 was used (white color), and RNAscope probes were used against LNC02446 (green) and HLA-DRB1 (magenta). Representative images from one patient per cohort are shown. An overlay image is shown at top, and individual channels are shown below. All slides passing quality control were quantitated (CD8: n=7 for active MC, n=7 for CD, n=9 for unaffected controls; LINC02446: n=6 for active MC, n=5 for CD, n=9 for unaffected controls; HLA-DRB1: n=4 for active MC, n=6 for CD, n=5 for unaffected controls) and proportions of positive cells were plotted as boxplots (at right). An asterisk (*) indicates MC is significantly different from both controls at an FDR level of 0.05.

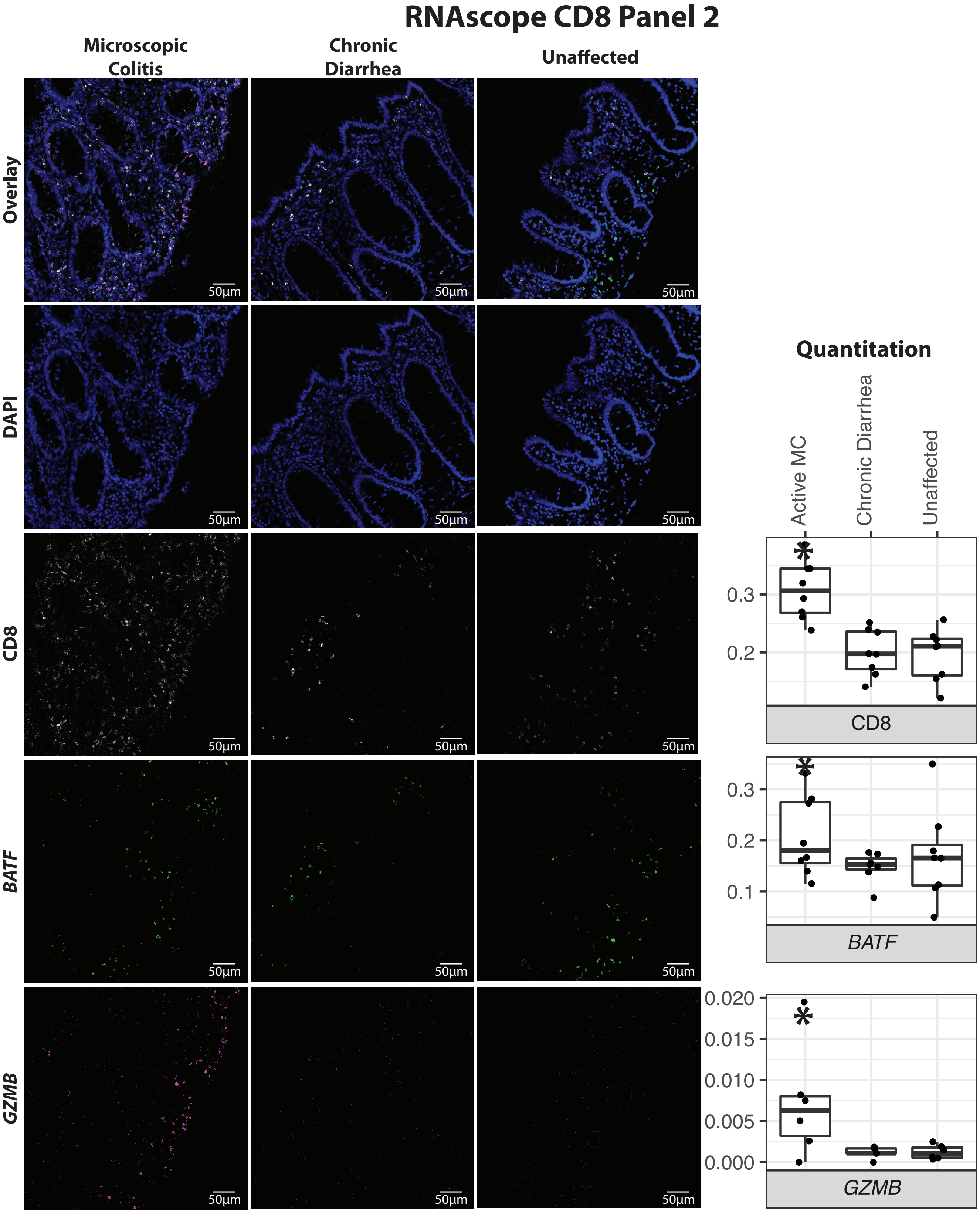

Supplementary Figure 11

A.) Patient biopsies were stained using RNAscope technology and imaged with confocal microscopy. An antibody against CD8 was used (white color), and RNAscope probes were used against BATF (green) and GZMB (magenta). Representative images from one patient per cohort are shown. An overlay image is shown at top, and individual channels are shown below. All slides passing quality control were quantitated (CD8: n=8 for active MC, n=8 for CD, n=8 for unaffected controls; BATF: n=8 for active MC, n=7 for CD, n=8 for unaffected controls; GZMB: n=6 for active MC, n=5 for CD, n=6 for unaffected controls) and proportions of positive cells were plotted as boxplots (at right). An asterisk (*) indicates MC is significantly different from both controls at an FDR level of 0.05.

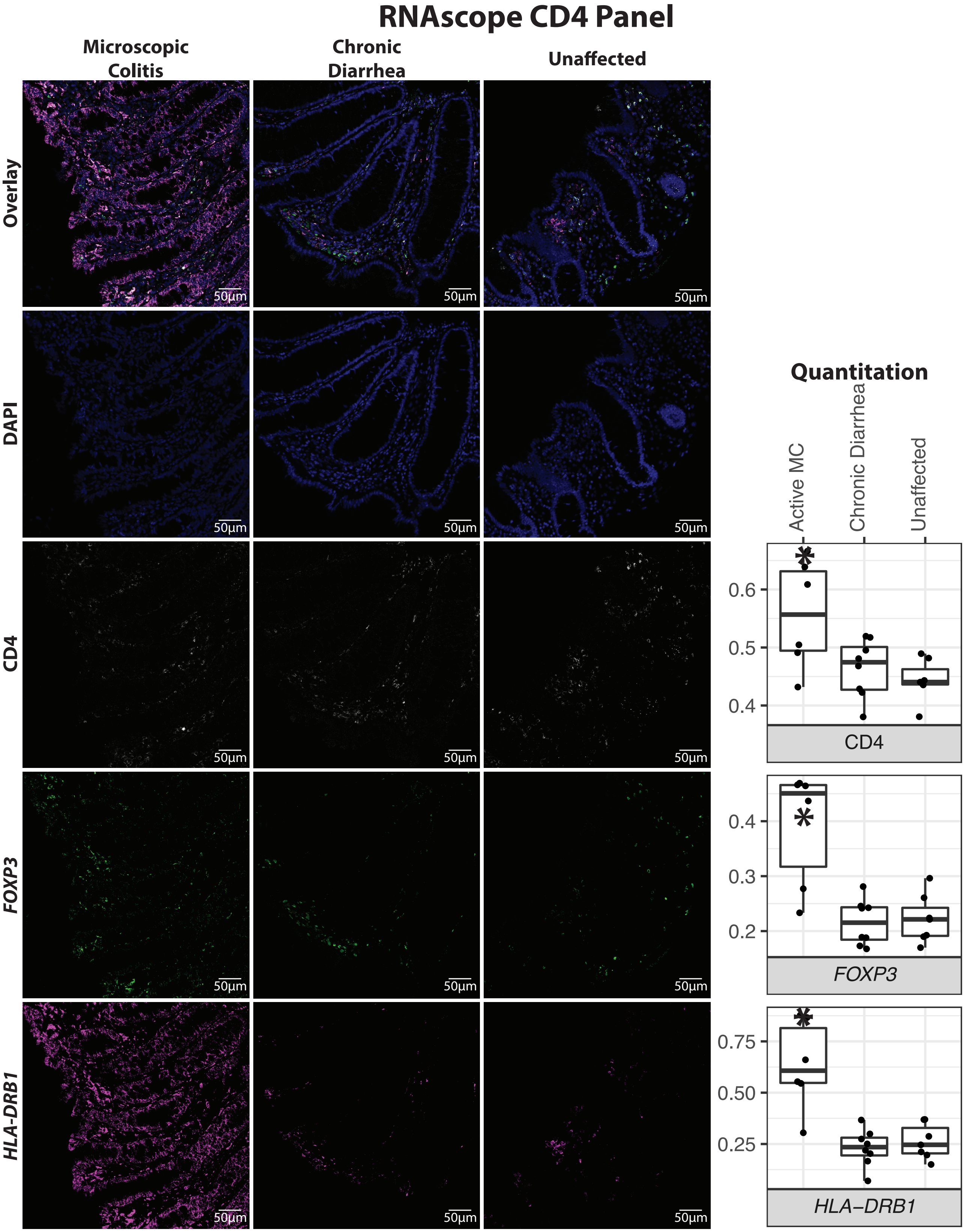

Supplementary Figure 12
A.) Patient biopsies were stained using RNAscope technology and imaged with confocal microscopy. An antibody against CD4 was used (white color), and RNAscope probes were used against FOXP3 (green) and HLA-DRB1 (magenta). Representative images from one patient per cohort are shown. An overlay image is shown at top, and individual channels are shown below. All slides passing quality control were quantitated (n=6 for active MC, n=8 for CD, n=7 for unaffected controls) and proportions of positive cells were plotted as boxplots (at right). An asterisk (*) indicates MC is significantly different from both controls at an FDR level of 0.05.

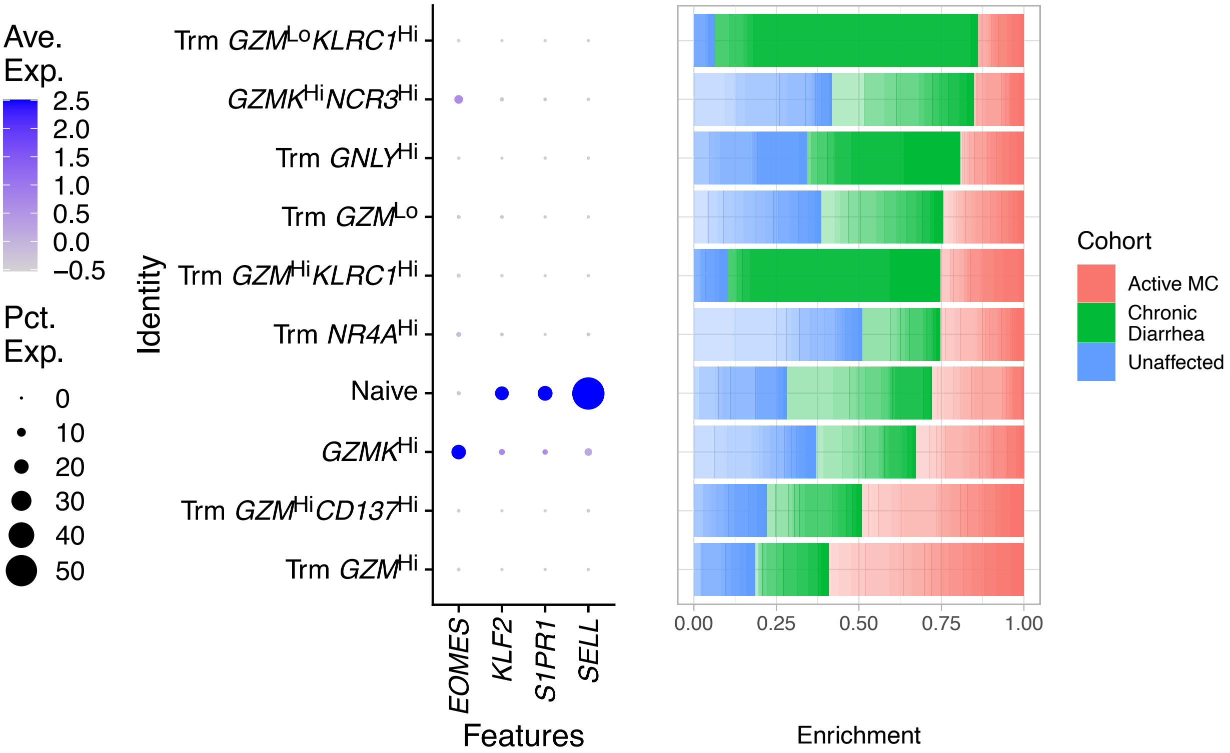

Supplementary Figure 13

Expression of genes associated with circulatory and recirculating T cells. CD8 T cells were examined for signs of circulation, and only Naïve cells presented with high expression of circulatory markers. GZMKHi T cells expressed EOMES, characteristic of recirculating T cells. A Stacked bar plot demonstrating the relative enrichment of each cell subset by cohort designation is shown at the right. The GZMKHi T cells do not expand in MC.

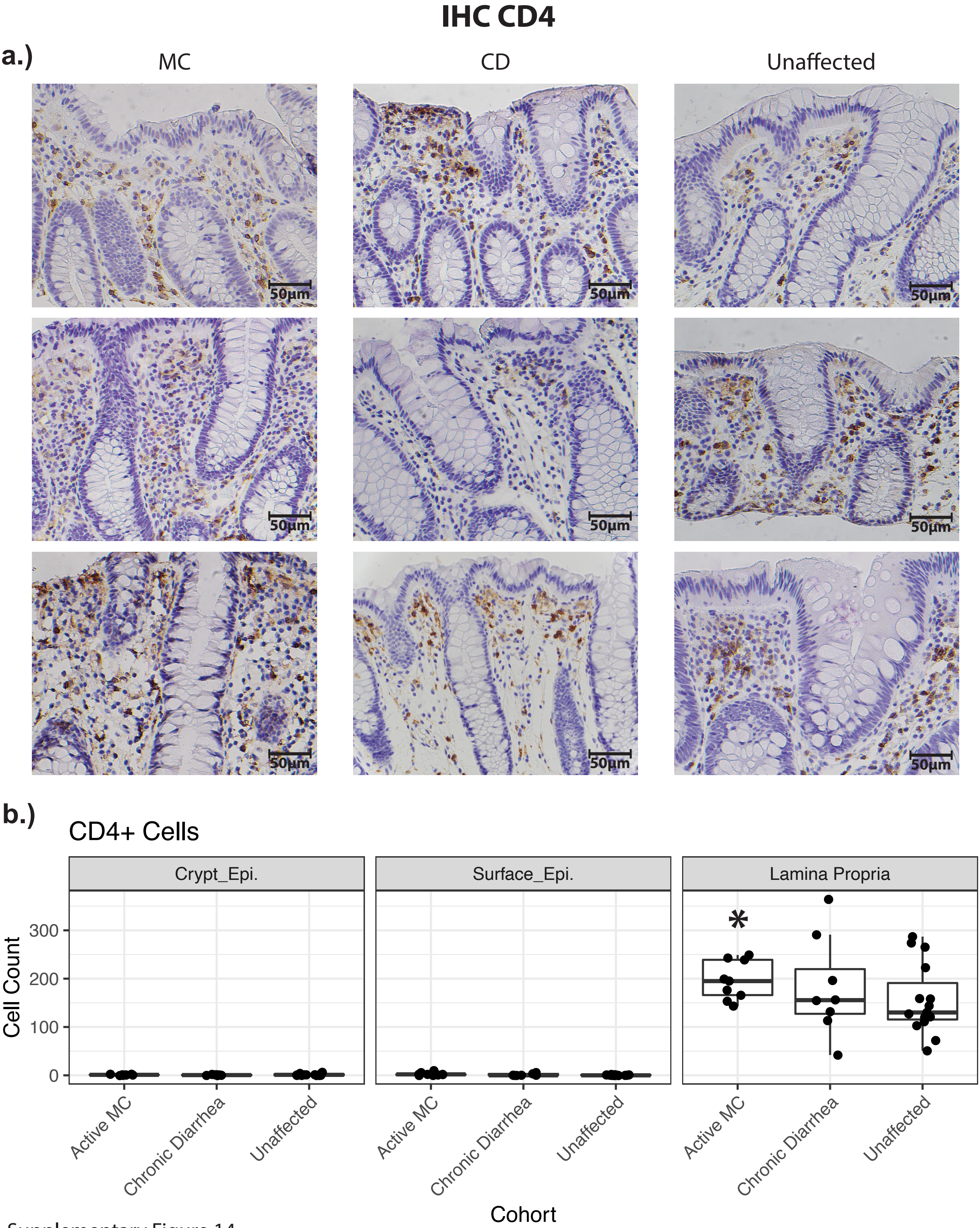

Supplementary Figure 14

A.) Patient biopsies were stained for CD4 using IHC. Representative images from three patients per cohort are shown. B.) Positive cells from all patient slides (n=9 for active MC, n=8 for chronic diarrhea, and n=15 for unaffected controls) were quantitated separately in the crypt epithelial cells, apical epithelial cells, and lamina propria. Cell counts for all slides are plotted as boxplots. An asterisk (*) indicates MC is significantly different from both controls at an FDR level of 0.05.

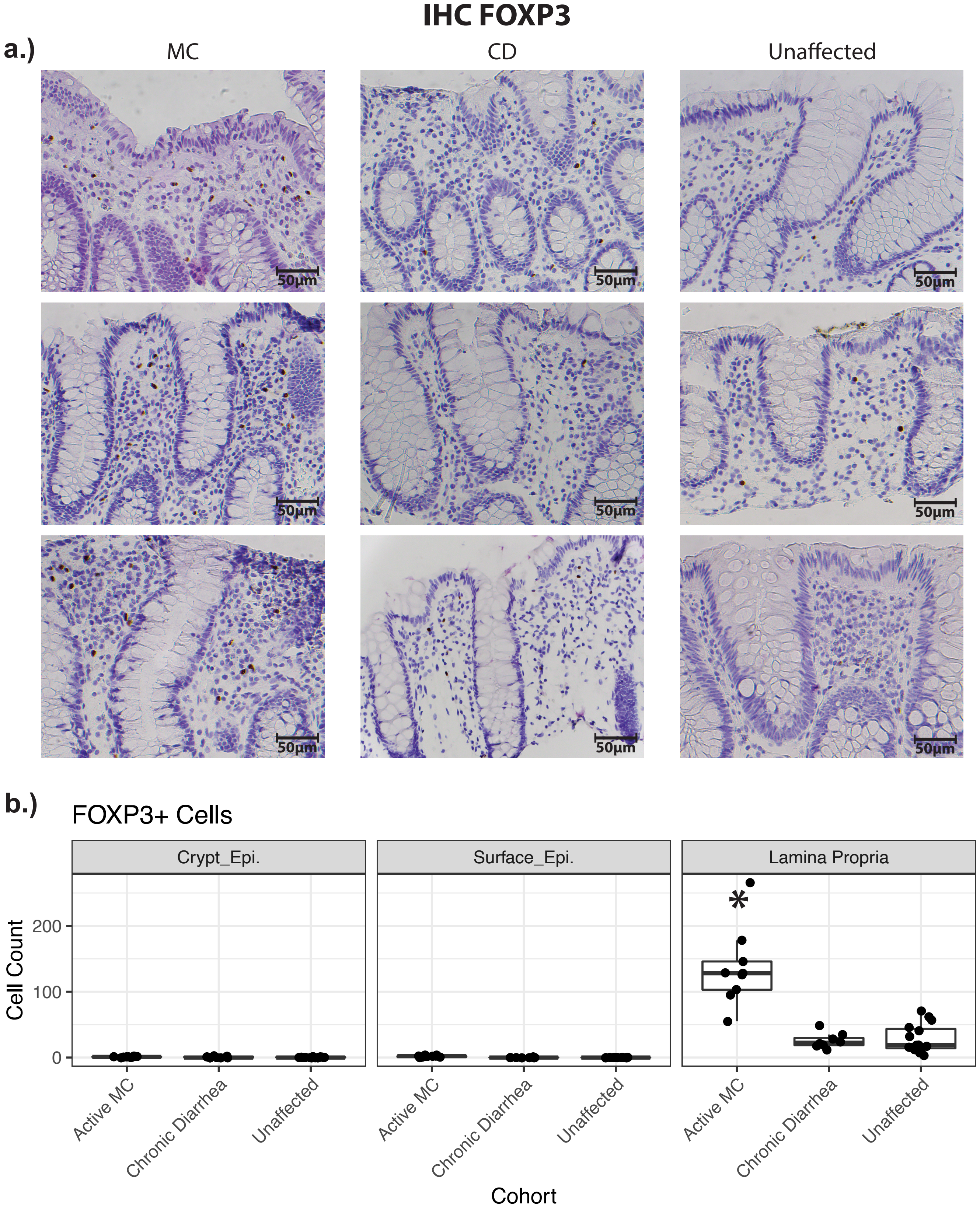

Supplementary Figure 15
A.) Patient biopsies were stained for FOXP3 using IHC. Representative images from three patients per cohort are shown. B.) Positive cells from all patient slides (n=9 for active MC, n=8 for chronic diarrhea, and n=15 for unaffected controls) were quantitated separately in the crypt epithelial cells, apical epithelial cells, and lamina propria. Cell counts for all slides are plotted as boxplots. An asterisk (*) indicates MC is significantly different from both controls at an FDR level of 0.05.

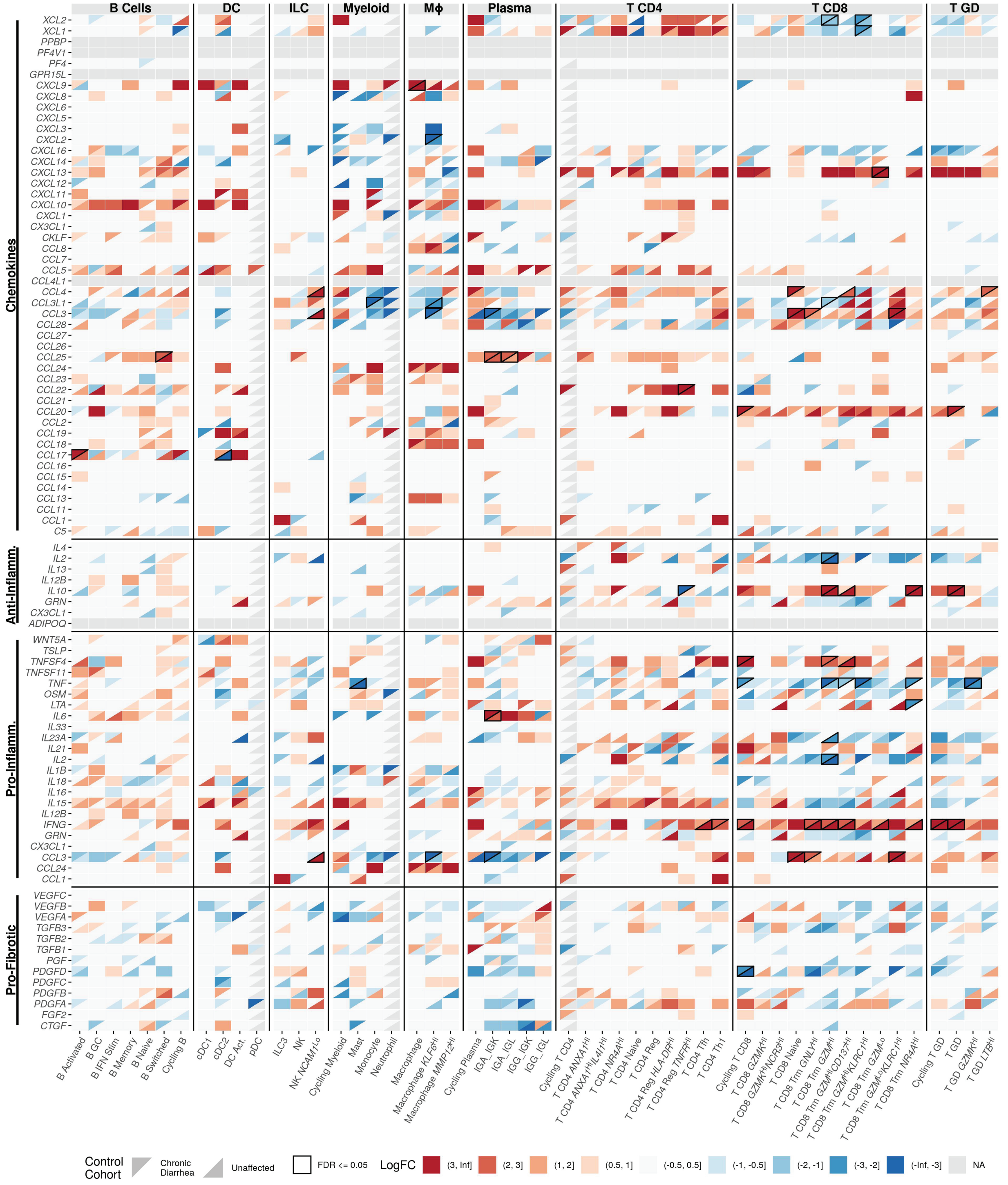

Supplementary Figure 16

Gene expression comparison heatmaps, as in Figure 6A. Here, all the proinflammatory cytokines, anti-inflammatory cytokines, chemokines, and a selection of profibrotic factors are plotted in heatmaps.

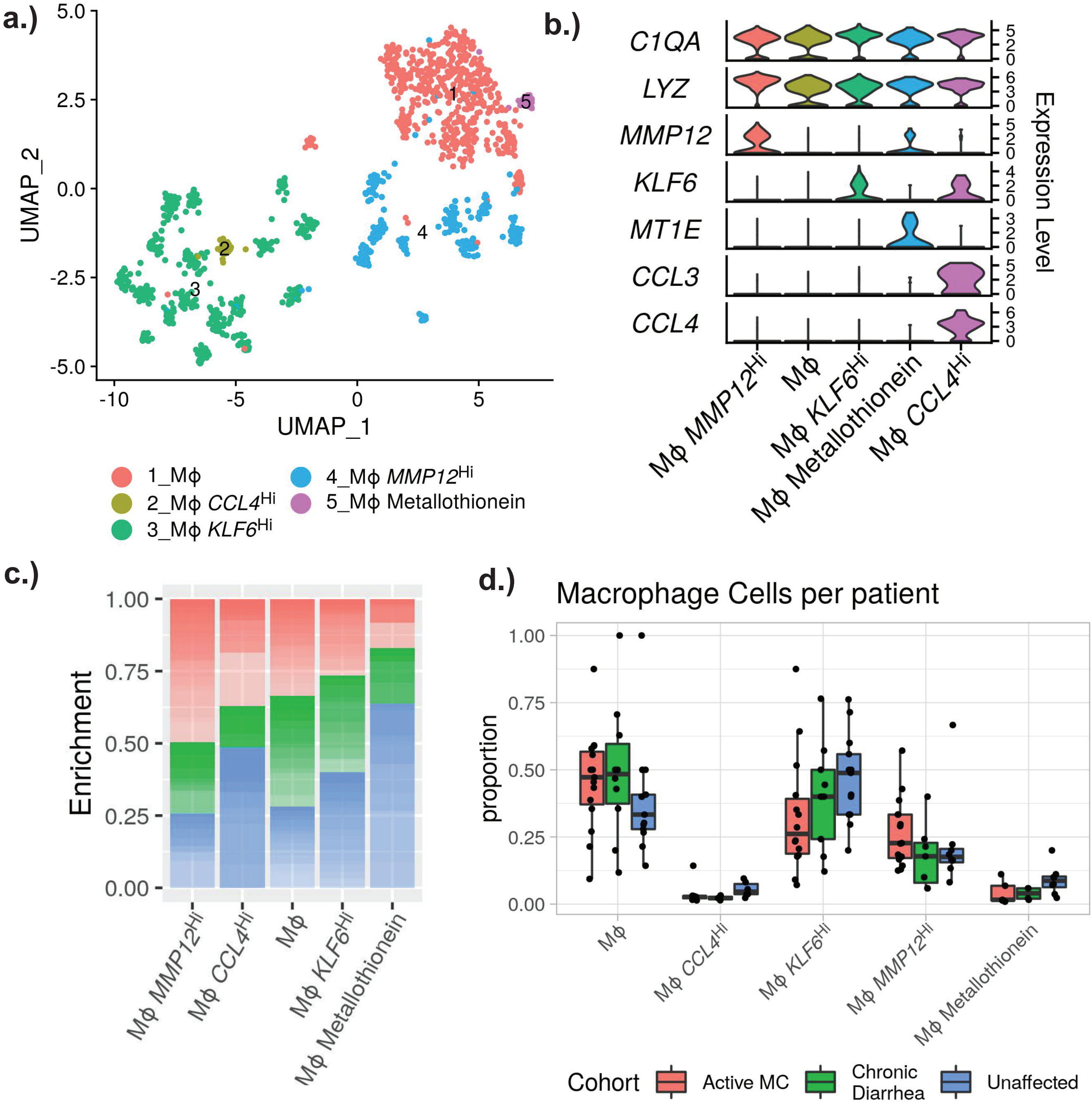

Supplementary Figure 17

A.) UMAP plot showing the subclustering of all macrophage cells, and corresponding cluster designations. B.) Violin plots showing the distribution of representative markers for the identified macrophage subtypes. C.) Stacked bar plot demonstrating the relative enrichment of each macrophage cell subset by cohort designation. D.) Boxplots showing the distribution of subtypes of macrophages.

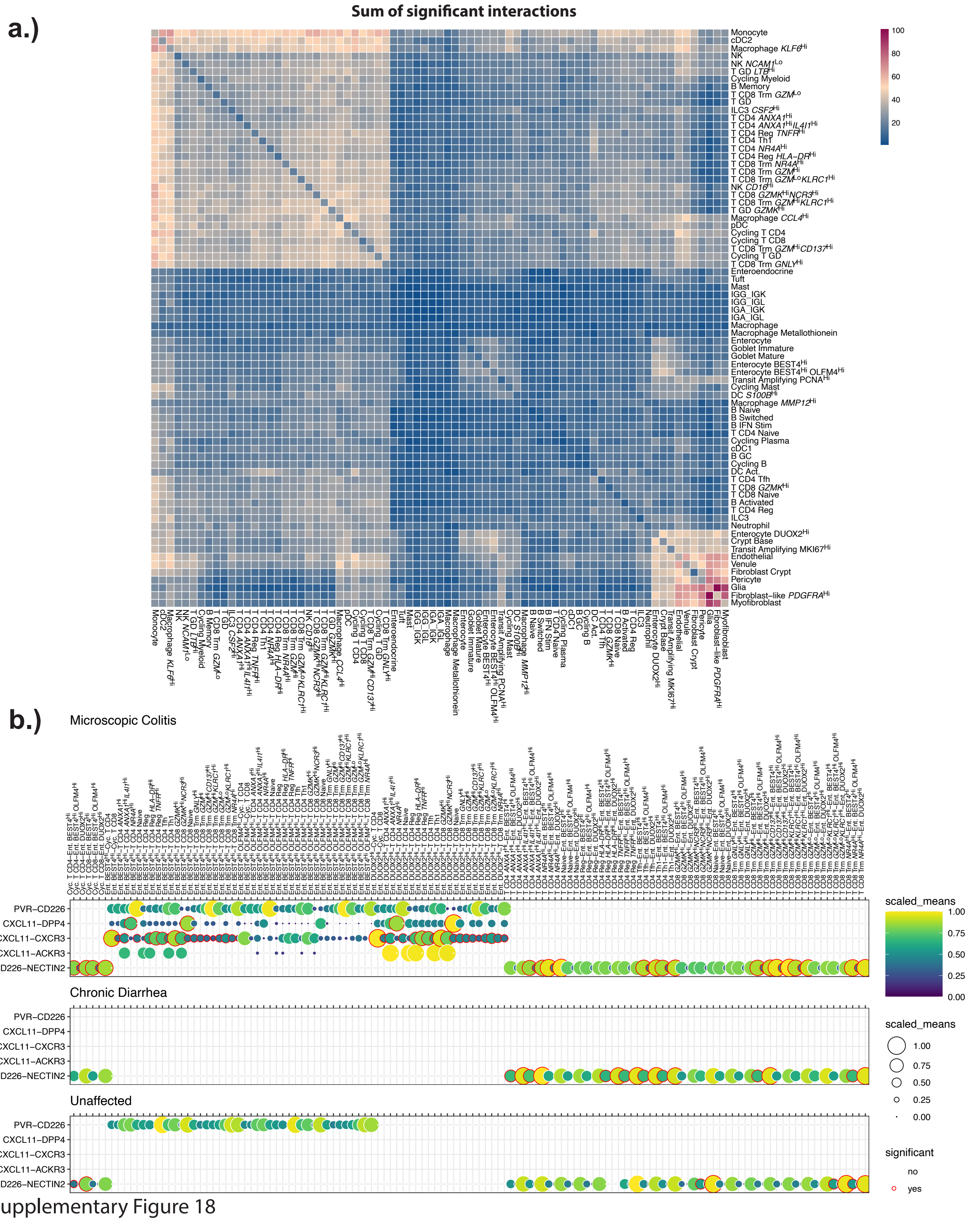

Supplementary Figure 18

A.) CellPhoneDB interaction heatmap showing the sum of significant interactions between all cell types (including immune, epithelial, and stromal cells). There is little cross-talk between major cell compartments, with most potential signaling events contained within a specific compartment. B.) Differential cross-compartment signaling between MC and controls was most pronounced between T cells and enterocytes, with significant differences in CXCL11 and CD226 signaling events. CXCL11 and CD226 interaction scores are plotted separately for MC (top), chronic diarrhea (middle), and unaffected controls (bottom).

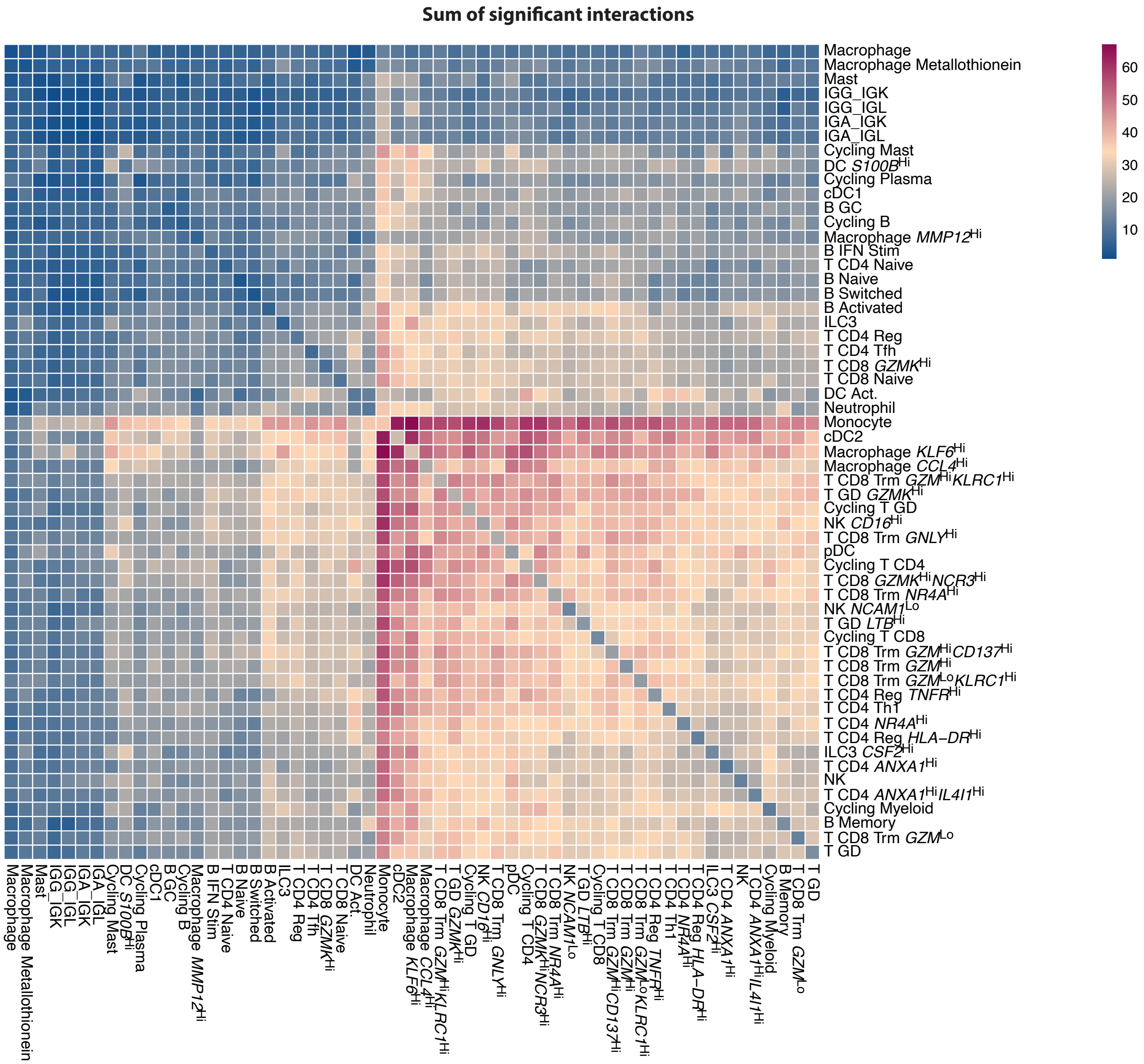

Supplementary Figure 19

CellPhoneDB interaction heatmap showing the sum of significant interactions between only the subtypes of immune cells. The cells with the highest potential for interactions include T cells and subtypes of myeloid cells.

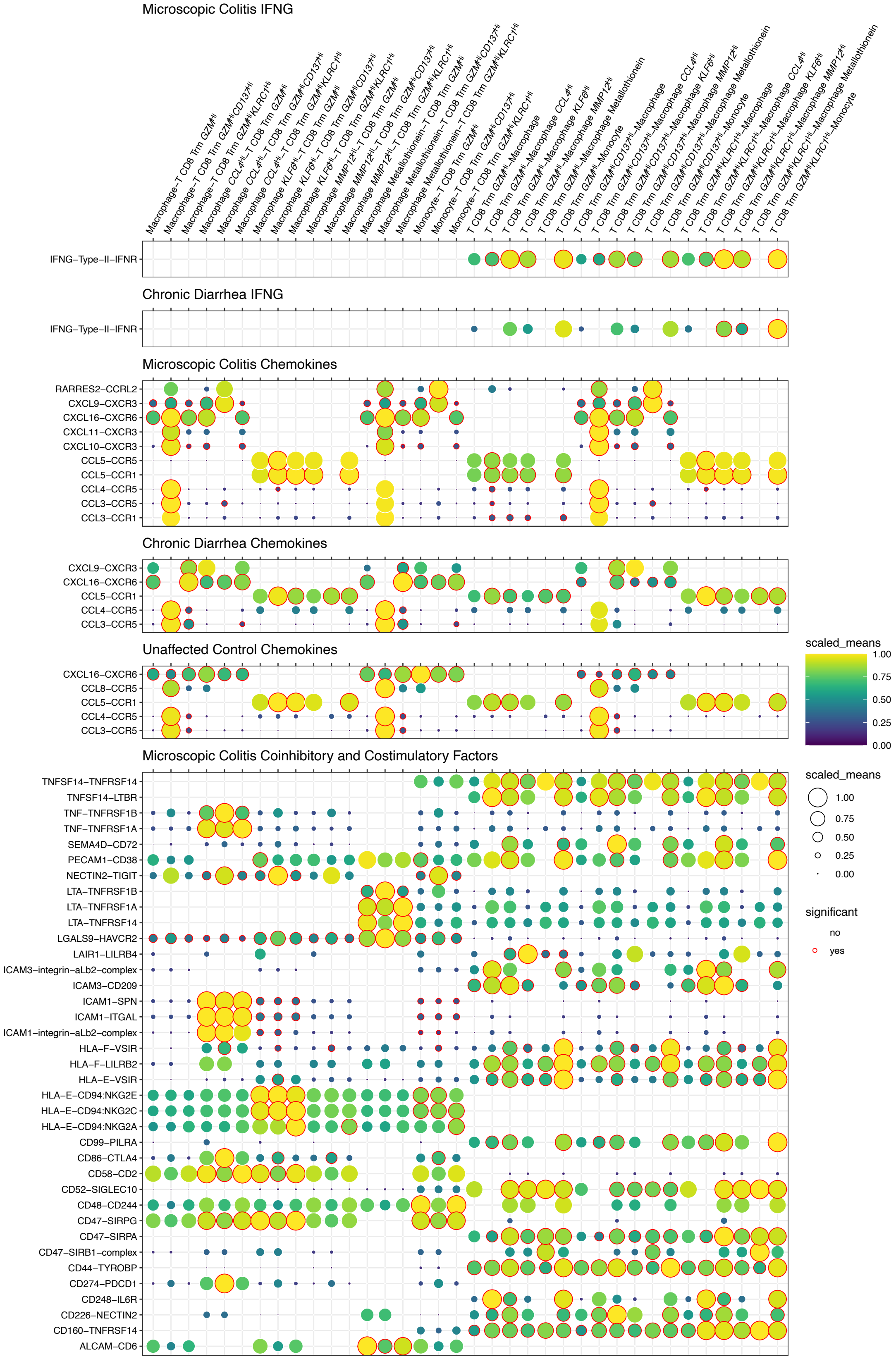

Supplementary Figure 20

CellPhoneDB receptor-ligand interactions plotted as a dot plot. Significant interactions for IFNG between CD8 T cells and Macrophages are plotted separately for MC and chronic diarrhea samples. Unaffected control samples presented no significant interactions through IFNG and were omitted from this plot. All significant chemokine interactions are plotted separately for MC, chronic diarrhea, and unaffected controls. A variety of coinhibitory and costimulatory receptor-ligand pairs (bottom) is plotted for MC.

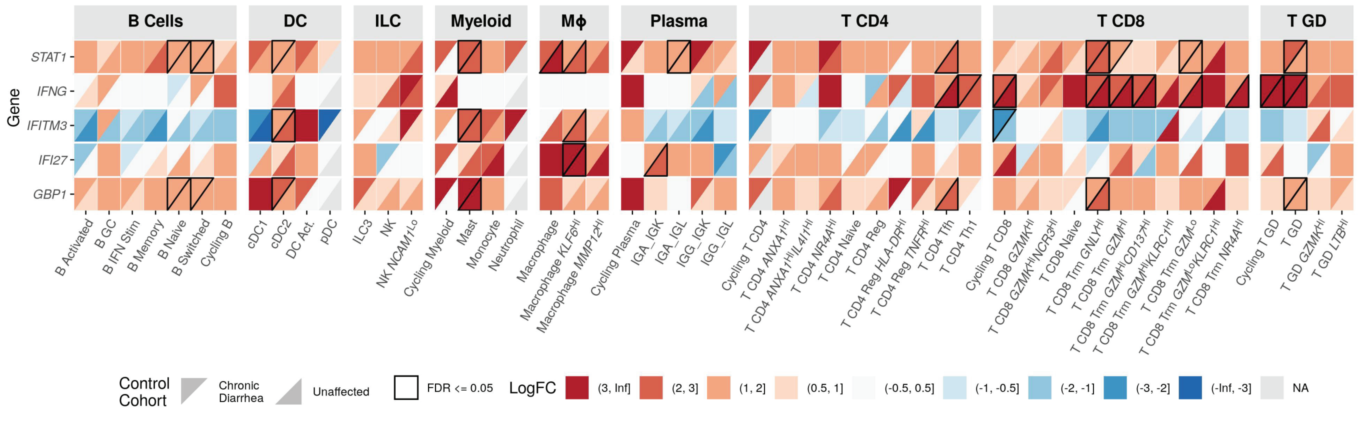

Supplementary Figure 21

scRNAseq data was examined for downstream signs of interferon stimulation in MC patients. IFNG as well as a selection of genes upregulated after interferon stimulation are plotted as a heatmap, with similar layout as in Figure 6A. IFNG is upregulated predominantly in CD8 T cells, while myeloid cells exhibit the strongest signs of downstream IFN signalling.

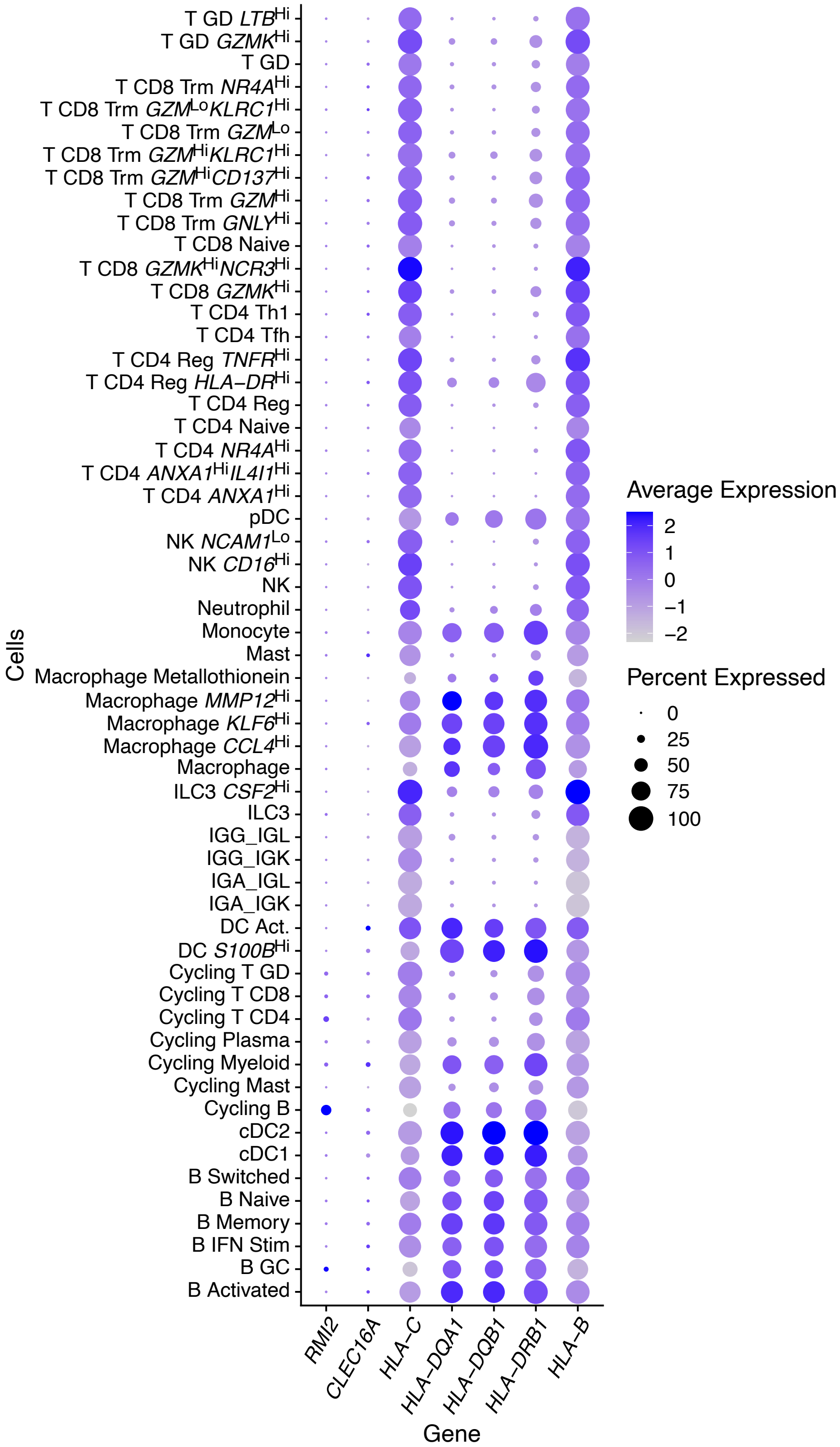

Supplementary Figure 22
Expression of genes previously associated with MC based on GWAS studies. The MHC-II gene HLA-DRB1 was previously found to have the strongest associated with MC, while only modest associations were identified for the other genes presented. MHC-II genes are found at the highest levels in antigen presenting cells. The MC-associated changes in expression for each of these genes is shown in Figure 6A.

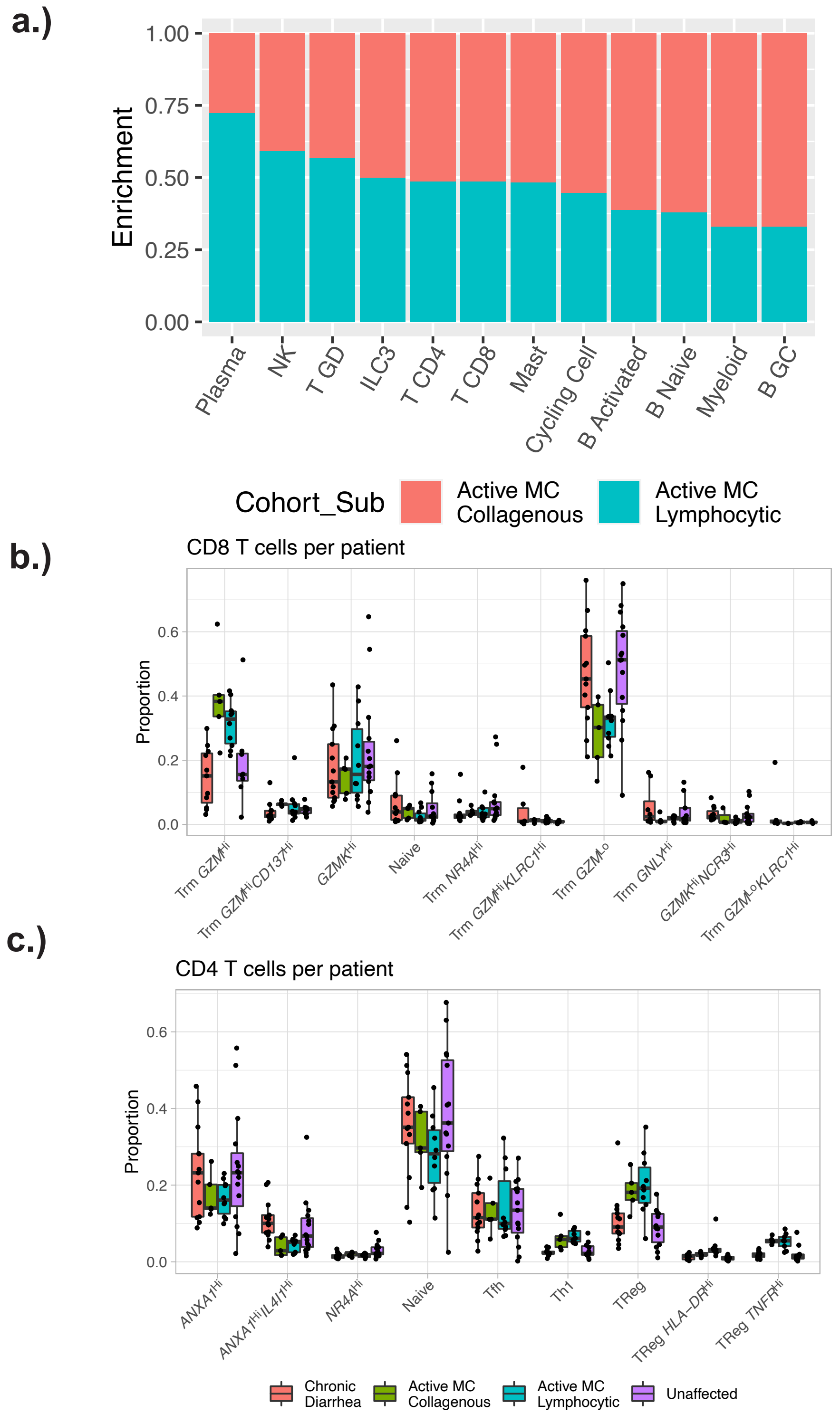

Supplementary Figure 23

A.) Stacked bar plot illustrating the enrichment of different cell types between the subtypes of MC: Lymphocytic and Collagenous colitis. An enrichment value of 0.5 indicates that Lymphocytic and Collagenous colitis present with similar proportions of that particular cell type. B.) Boxplots showing the per-patient proportional differences of the CD8+ T cell subtypes, grouped by cohort. Here, Lymphocytic and Collagenous colitis are considered as separate cohorts. The proportions shown are relative to the number of all CD8 T cells for each patient. Both Lymphocytic and Collagenous colitis present with similar cellular profiles. C.) Boxplots showing the per-patient proportional differences of the CD4 T cell subtypes, grouped by cohort. Here, Lymphocytic and Collagenous colitis are considered as separate cohorts. The proportions shown are relative to the number of all CD4 T cells for each patient. Both Lymphocytic and Collagenous colitis present with similar cellular profiles.

B Cells

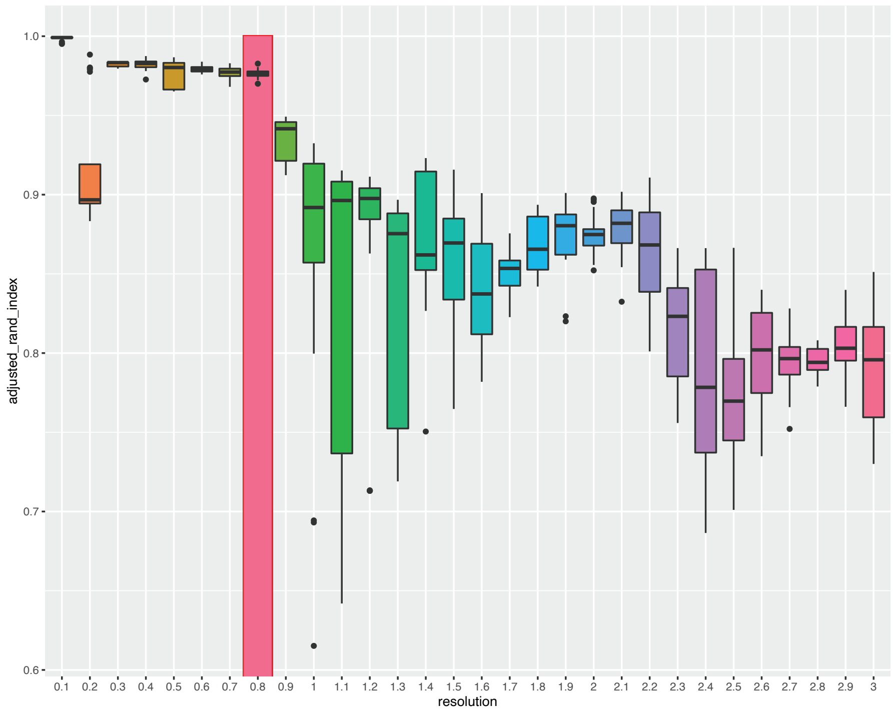

CD8 T Cells

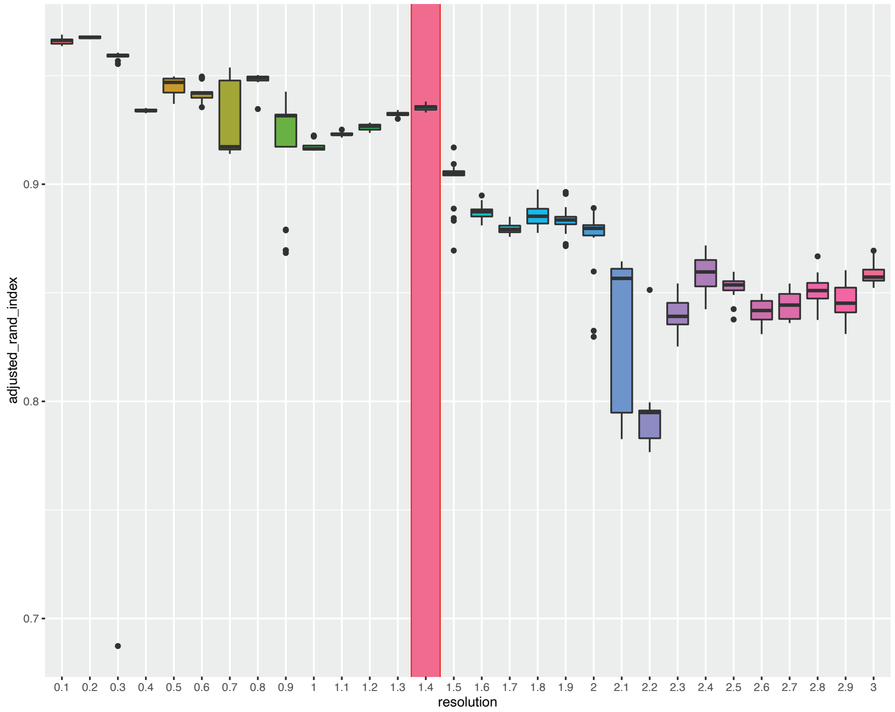

Supplementary Figure 24
Boxplots illustrating the process for selecting optimal clustering resolution. Adjusted rand index was plotted for each tested clustering resolution, and the highest resolution before the rand index exhibited a strong decrease was selected for downstream analysis. Representative examples of this process are shown for B and T cells.
