# Supplementary material for: Single-cell transcriptomic characterization of microscopic colitis
Source: Nat Commun. 2025 May 18;16:4618. doi: 10.1038/s41467-025-59648-8 (PMC12086216; doi:10.1038/s41467-025-59648-8)
Supplement: Supplementary file 2 — Description of Additional Supplementary Files [file 41467_2025_59648_MOESM2_ESM.pdf]

Description of Additional Supplementary Files

Supplementary Data 1

Metadata for each patient in the final dataset. Note that one patient was dropped during quality control, bringing the final patient count to 45. MC_SUBTYPE indicates the clinical subtype of diagnosed Microscopic Colitis (LC=Lymphocytic Colitis; CC=Collagenous Colitis; LC_CC=Indications of both Lymphocytic and Collagenous; NA=Not Applicable; UNK=Subtype not listed in patient file). AGE represents patient age (in years) at time of biopsy collection. RACE indicates patient-reported race (0=Non-white; 1=White; 2=Not Reported). SEX indicates patient-reported biological sex (0=Male; 1=Female). EVER_SMOKER indicates patient-reported history of smoking (0=Non-smoker; 1=Past or present smoker). BMI indicates patient body mass index at time of biopsy (UNK=not recorded).

Supplementary Data 2

Gene expression markers for each of the cell clusters. In this table, “subclust_level” indicates the subclustering level for each comparison. A level of 0 indicates the cluster was compared against all other immune/stromal/epithelial cells, a level of 1 indicates the cluster was compared against other B cells, T cells, etc. The specific reference population each cluster was compared against is listed in the “ref_cells” column. A Wilcoxon-Rank-Sum test was used to compute p values. P values were then adjusted for multiple hypothesis testing (p_val_adj column).

Supplementary Data 3

Pseudo-bulk style differential expression analysis was used to compare cells from patients with MC against patients with chronic diarrhea or unaffected controls. This table shows the differentially expressed genes in each cluster. Here, the “comp” column refers to which cohort was used as the reference control (CD=chronic diarrhea; H=unaffected controls). This table has been filtered to only include genes with an adjusted p-value (FDR correction) less than 0.1.
