# Supplementary material for: Single-cell transcriptomic characterization of microscopic colitis
Source: Nat Commun. 2025 May 18;16:4618. doi: 10.1038/s41467-025-59648-8 (PMC12086216; doi:10.1038/s41467-025-59648-8)
Supplement: Supplementary file 6 — Reporting Summary [file 41467_2025_59648_MOESM6_ESM.pdf]

nature portfolio

Corresponding author(s): Hamed Khalili

Last updated by author(s): Jan 25, 2025

Reporting Summary

Nature Portfolio wishes to improve the reproducibility of the work that we publish. This form provides structure for consistency and transparency in reporting. For further information on Nature Portfolio policies, see our Editorial Policies and the Editorial Policy Checklist.

Statistics

For all statistical analyses, confirm that the following items are present in the figure legend, table legend, main text, or Methods section.

n/a	Confirmed	
☐	☒	The exact sample size (*n*) for each experimental group/condition, given as a discrete number and unit of measurement
☐	☒	A statement on whether measurements were taken from distinct samples or whether the same sample was measured repeatedly
☐	☒	The statistical test(s) used AND whether they are one- or two-sided<br>*Only common tests should be described solely by name; describe more complex techniques in the Methods section.*
☐	☒	A description of all covariates tested
☐	☒	A description of any assumptions or corrections, such as tests of normality and adjustment for multiple comparisons
☐	☒	A full description of the statistical parameters including central tendency (e.g. means) or other basic estimates (e.g. regression coefficient) AND variation (e.g. standard deviation) or associated estimates of uncertainty (e.g. confidence intervals)
☐	☒	For null hypothesis testing, the test statistic (e.g. *F*, *t*, *r*) with confidence intervals, effect sizes, degrees of freedom and *P* value noted<br>*Give P values as exact values whenever suitable.*
☒	☐	For Bayesian analysis, information on the choice of priors and Markov chain Monte Carlo settings
☒	☐	For hierarchical and complex designs, identification of the appropriate level for tests and full reporting of outcomes
☒	☐	Estimates of effect sizes (e.g. Cohen's *d*, Pearson's *r*), indicating how they were calculated

Our web collection on statistics for biologists contains articles on many of the points above.

Software and code

Policy information about availability of computer code

Data collection	Data were generated using Illumina HiSeq and NextSeq instruments.
Data analysis	Data analysis was performed with the software and versions described in the methods section. Custom processing scripts for modifying read format have been submitted to the Zenodo repo listed. R code for generating all the figure panels is also in the Zenodo repo.

For manuscripts utilizing custom algorithms or software that are central to the research but not yet described in published literature, software must be made available to editors and reviewers. We strongly encourage code deposition in a community repository (e.g. GitHub). See the Nature Portfolio guidelines for submitting code & software for further information.

Data

Policy information about availability of data

All manuscripts must include a data availability statement. This statement should provide the following information, where applicable:

- Accession codes, unique identifiers, or web links for publicly available datasets
- A description of any restrictions on data availability
- For clinical datasets or third party data, please ensure that the statement adheres to our policy

The sequencing data generated in this study have been deposited in the dbGaP database under accession code phs003876.v1.p1. The sequencing data are available under restricted access as per our IRB protocol. Access can be obtained by submitting a Data Use Certificate to the NIH Data Access Committee for approval. The processed data are available at Zenodo with DOI 10.5281/zenodo.14661972

Research involving human participants, their data, or biological material

Policy information about studies with human participants or human data. See also policy information about sex, gender (identity/presentation), and sexual orientation and race, ethnicity and racism.

Reporting on sex and gender	Self-reported sex information was collected from every participant, and is outlined in Fig. 1. The majority of subjects in this dataset are female. This mirrors the significantly higher prevalence of microscopic colitis in female that has been widely reported in other studies. Our chronic diarrhea and unaffected control cohorts were biased towards females to match the sex distribution in patients with microscopic colitis.
Reporting on race, ethnicity, or other socially relevant groupings	Self-reported race and smoking status was collected from every participant, and is outlined in Fig. 1.
Population characteristics	Our three cohorts were age-matched to the best of our ability. The mean age of microscopic colitis patients was 66.8 years, unaffected control patients 64.5 years, and chronic diarrhea patients 57.0 years. All of the unaffected control patients were of white race, 92% of chronic diarrhea patients were of white race, and 88% of microscopic colitis patients were of white race. The mean BMI was 26.9 kg/m2 for the microscopic colitis group, 28.4 kg/m2 for the unaffected controls, and 30.3 kg/m2 for patients with chronic diarrhea. The prevalence of self-reported history of smoking (past or current) were 44% in the microscopic colitis cohort , 31% in the unaffected control cohort, and 62% in the chronic diarrhea cohort.
Recruitment	Subjects were recruited from the patient population receiving care in the MGH Gastroenterology Unit. Patients with suspected or established microscopic colitis who were scheduled to receive a diagnostic colonoscopy or attend a clinic visit were recruited, along with healthy patients who were scheduled to receive a screening colonoscopy.
Ethics oversight	The institutional review board at Mass General Brigham and Dana-Farber approved this study (MGB protocols 2015P001333 and 2015P000275, DFCI/HCC protocols 11-181 and 13-416).

Note that full information on the approval of the study protocol must also be provided in the manuscript.

Field-specific reporting

Please select the one below that is the best fit for your research. If you are not sure, read the appropriate sections before making your selection.

[x] Life sciences [ ] Behavioural & social sciences [ ] Ecological, evolutionary & environmental sciences

For a reference copy of the document with all sections, see nature.com/documents/nr-reporting-summary-flat.pdf

Life sciences study design

All studies must disclose on these points even when the disclosure is negative.

Sample size	Due to budgetary restrictions, we were limited to profiling a total of 46 patients. The tools we used for compositional analysis takes sample size into account when calculating p-values.
Data exclusions	Most single cell analyses remove cells with an apoptotic signature, or those that contain especially high proportions of mitochondrial genes, indicative of cell death. We have done the same, and detailed this in our methods section. We additionally removed one patient's cells from the dataset because of data quality issues -- there were far more cells than expected, and most of the cells contained multiple markers from distant lineages; these two signs strongly implicate cell overloading during the encapsulation phase (which would cause most of the droplets to contain multiple cells), so we removed this patient's cells from downstream analysis.
Replication	We did not replicate the single-cell RNAseq experiments due to budgetary and time considerations; we only have one set of patients and analysis, without a follow up "Validation" cohort. We validated our central findings using two histological techniques, immunohistochemistry and RNAscope.
Randomization	Participants were assigned to cohorts based on the clinical symptoms (normal bowel movements vs. chronic diarrhea) as well as the microscopic characterization of their biopsies (over or under 20 IELs per 100 epithelial cells). This is outlined in Figure 1.
Blinding	At the time of biopsy collection and processing, the doctor performing the biopsy was aware of the clinical symptoms, but not the diagnosis. The research coordinator performing the tissue dissociation was also aware of the clinical symptoms but not the diagnosis. The personnel performing the cell encapsulation were not aware of the grouping.

Reporting for specific materials, systems and methods

We require information from authors about some types of materials, experimental systems and methods used in many studies. Here, indicate whether each material, system or method listed is relevant to your study. If you are not sure if a list item applies to your research, read the appropriate section before selecting a response.

Materials & experimental systems

n/a	Involved in the study
☐	☒ Antibodies
☒	☐ Eukaryotic cell lines
☒	☐ Palaeontology and archaeology
☒	☐ Animals and other organisms
☒	☐ Clinical data
☒	☐ Dual use research of concern
☒	☐ Plants

Methods

n/a	Involved in the study
☒	☐ ChIP-seq
☒	☐ Flow cytometry
☒	☐ MRI-based neuroimaging

Antibodies

Antibodies used

ThermoFisher 14-2444-82 (dilution 1:100, clone N1UG0); Leica PA0183 (ready to use, no dilution, clone 4B11); ThermoFisher A-21037 (dilution 1:400)

Validation

CD4 monoclonal antibody has been used in 6 publications; CD8 antibody has regulatory approval for in vitro diagnostic use, and is used by the MGH clinical pathology core for patient samples; Goat anti-mouse IgG has been used in 48 publications

Plants

Seed stocks

Report on the source of all seed stocks or other plant material used. If applicable, state the seed stock centre and catalogue number. If plant specimens were collected from the field, describe the collection location, date and sampling procedures.

Novel plant genotypes

Describe the methods by which all novel plant genotypes were produced. This includes those generated by transgenic approaches, gene editing, chemical/radiation-based mutagenesis and hybridization. For transgenic lines, describe the transformation method, the number of independent lines analyzed and the generation upon which experiments were performed. For gene-edited lines, describe the editor used, the endogenous sequence targeted for editing, the targeting guide RNA sequence (if applicable) and how the editor was applied.

Authentication

Describe any authentication procedures for each seed stock used or novel genotype generated. Describe any experiments used to assess the effect of a mutation and, where applicable, how potential secondary effects (e.g. second site T-DNA insertions, mosiacism, off-target gene editing) were examined.
